# FBXL17/spastin axis as a novel therapeutic target of hereditary spastic paraplegia

**DOI:** 10.1186/s13578-022-00851-1

**Published:** 2022-07-22

**Authors:** Hyun Mi Kang, Dae Hun Kim, Mijin Kim, Yoohong Min, Bohyeon Jeong, Kyung Hee Noh, Da Yong Lee, Hyun-Soo Cho, Nam-Soon Kim, Cho-Rok Jung, Jung Hwa Lim

**Affiliations:** 1 Korea Research Institute of Bioscience and Biotechnology (KRIBB), 125 Gwahak-ro, 34141 Daejeon, Republic of Korea; 2 Department of Functional Genomics, Korea University of Science and Technology (UST), 217 Gajeong-ro, Daejeon, Republic of Korea; 3 Department of Microbiology, Chungbuk National University, 28644 Chungbuk, Republic of Korea; 4 Department of Biology, Chungnam National University, 34134 Daejeon, Republic of Korea

**Keywords:** SPAST, E3 ubiquitin ligase, SCF complex, FBXL17, Hereditary spastic paraplegia

## Abstract

**Background:**

Spastin significantly influences microtubule regulation in neurons and is implicated in the pathogenesis of hereditary spastic paraplegia (HSP). However, post-translational regulation of the spastin protein remains nebulous. The association between E3 ubiquitin ligase and spastin provides a potential therapeutic strategy.

**Results:**

As evidenced by protein chip analysis, FBXL17 inversely correlated with SPAST-M1 at the protein level in vitro and*,* also in vivo during embryonic developmental stage. SPAST-M1 protein interacted with FBXL17 specifically via the BTB domain at the N-terminus of SPAST-M1. The SCF^FBXL17^ E3 ubiquitin ligase complex degraded SPAST-M1 protein in the nuclear fraction in a proteasome-dependent manner. SPAST phosphorylation occurred only in the cytoplasmic fraction by CK2 and was involved in poly-ubiquitination. Inhibition of SCF^FBXL17^ E3 ubiquitin ligase by small chemical and FBXL17 shRNA decreased proteasome-dependent degradation of SPAST-M1 and induced axonal extension. The SPAST Y52C mutant, harboring abnormality in BTB domain could not interact with FBXL17, thereby escaping protein regulation by the SCF^FBXL17^ E3 ubiquitin ligase complex, resulting in loss of functionality with aberrant quantity. Although this mutant showed shortening of axonal outgrowth, low rate proliferation, and poor differentiation capacity in a 3D model, this phenotype was rescued by inhibiting SCF^FBXL17^ E3 ubiquitin ligase.

**Conclusions:**

We discovered that a novel pathway, FBXL17-SPAST was involved in pathogenicity of HSP by the loss of function and the quantitative regulation. This result suggested that targeting FBXL17 could provide new insight into HSP therapeutics.

**Supplementary Information:**

The online version contains supplementary material available at 10.1186/s13578-022-00851-1.

## Introduction

Spastin, encoded by the *SPAST* gene, harbors a microtubule (MT)-interacting and trafficking domain, and an ATPase, with diverse cellular activities (AAA), domain; hence, it plays a critical role in regulating MT dynamics [1]. Wild-type SPAST severs MTs by destabilizing alpha- and beta-tubulin interactions and MT bundling in vitro, while acetylation of α-tubulin has been considered an indicator of MT stability [2]. Severing is essential for axonal MT transport, and the fine regulation of severing and bundling is essential for MT homeostasis. Thus, the loss of SPAST function induces axonal swelling and accumulation of micro-organelles in axonal fibers [3, 4]. The *SPAST* gene has two initiation sites, resulting in two major isoforms, namely the 616-amino-acid isoform, M1, and the N-terminal truncated 530-amino-acid isoform, M87. Both isoforms have AAA ATPase activity, but M1 performs major roles in neurons by regulating endoplasmic reticulum (ER) dynamics [5], while its mutation is neurotoxic [2] and accounts for > 40% of autosomal dominant (AD) forms of hereditary spastic paraplegias (HSPs) [6–8]. While the M87 isoform is widely and abundantly expressed due to the strong Kozak sequence, the expression of the M1 isoform is restricted by cell type and quantity in cells. Therefore, SPAST-M1 regulation is critical for understanding the pathophysiology and developing therapeutic strategies against HSPs [9]. Previously, we found that SPAST-M1 protein is regulated by the ubiquitin–proteasome pathway [10], which led to the screening of the SPAST-based E3 ubiquitin ligase, and the detection of FBXL17 (F-box and leucine-rich repeat protein 17) using a protein chip assay.

FBXL17 acts as an F-box protein of the E3 ubiquitin ligase complex, SCF (Skp1-Cul1-F-box protein), specifically targeting substrates by recognizing the F-box [11]. E3 ubiquitin ligase is sequentially activated by E1, a ubiquitin-activating enzyme, and E2, a ubiquitin transfer enzyme, and generates a polyubiquitin chain on the target for degradation in a proteasome-dependent manner [12]. Specificity is determined by the interaction between the F-box protein and the substrate, which possess diverse substrate recognition domains, such as FBXWs (WD repeats), FBXOs (other domains), and FBXLs (leucine-rich repeats). FBXLs recognize the substrate’s BTB domain, and members of the BTB domain proteins, including BACH1, KLHL3, and KEAP1, play critical roles in mammalian development and are associated with various diseases [13]. We uncovered that FBXL17 induced ubiquitin–proteasome-dependent spastin degradation and proposed that the spastin protein should be included as a new member of the BTB domain family. This finding may provide a novel mechanism for the development of novel therapeutic strategies.

## Methods

### Reagents and antibodies

All antibodies and reagents used in this study are listed in Additional file 2: Tables S1 and S2.

### Plasmid constructions and Lentivirus production

The SPAST and FBXL17 expression constructs were amplified using RT-PCR using cDNA and incorporated into various plasmid vectors. Detailed information on the plasmid constructs is provided in Additional file 2: Table S3. The 3xHA-FBXL17 clone was purchased from GeneCopoeia Inc. (EX-H4410-M06, Rockville, MD, US). For lentiviral vector construction and production, the lenti-FBXL17_shRNA clone was purchased from OriGene (#TL304572, Rockville, MD, US). For the production of Y52C-SPAST expressing lentivirus, 293FT cells were transfected with pLVx-Y52C SPAST along with psPAX2 (Genome), PMD2.G (Envelope), and then media was harvested 48 h post-transfection. For generation of CK2β-Cas9 stable cell, the CK2β CRISPR/Cas9 (sc-401684) and CK2β homology-induced repair (HDR, sc-401684-HDR) plasmids were purchased from Santa Cruz Biothechnology (Dallas, TX, US).

### Identification of the SPAST binding proteins through HuProt™ microarray

To identify SPAST binding proteins, His-tagged SPAST protein was induced and purified in an *Escherichia coli* expression system and then analyzed at Gene On Biotech (Daejeon, Republic of Korea) using Human Huprot™ protein microarray (CDI Labs, Mayaguez, PR, US). Human protein microarray, which contains over 20,000 full-length recombinant human proteins, was used. Briefly, the protein microarray was incubated with blocking buffer (2% BSA in 1 × PBS with 0.1% tween-20) for 2 h, and 3 μg of biotinylated His-SPAST was treated onto the array for 8 h at 4 ℃. Subsequently, the array was incubated with 1 μg of streptavidin-fluorescence (Alexa-Fluor 635 nm) for 1 h at 4 ℃. The microarray result was detected using a GenePix4100A microarray laser scanner (Molecular Devices, San Jose, CA, US).

### Cell culture

HEK293T and HeLa cells were maintained in Dulbecco’s modified Eagle’s medium (DMEM, welgene, Gyeongsan, Republic of Korea) with 10% fetal bovine serum (FBS, Thermo Fisher Scientific, Waltham, MA, US) and antibiotics (Thermo Fisher Scientific) in a humidified incubator with 5% CO_2_ at 37 °C. For proliferation, ReNcell CX was cultured in ReN NSC maintenance medium (Merck Millipore, Burlington, MA, US) with 20 ng/ml epidermal growth factor (EGF, Peprotech, Rocky Hill, NJ, USA), 20 ng/ml basic fibroblast growth factor (bFGF, Peprotech), and penicillin/streptomycin antibiotics. For the neuronal differentiation, ReNcell CX was differentiated in ReN NSC maintenance medium (SCM005, Merck Millipore) containing penicillin/streptomycin antibiotics without EGF and bFGF for the indicated days.

### Transfection

For transient transfection into HEK293 cells, cells were transfected with indicated plasmid constructs using Transporter™ 5 transfection reagent (Polysciences Inc. Warrington, PA, US). For the electroporation, ReNcell CX cells were harvested, resuspended in BTX electroporation buffer (BTX, Holliston, MA, US), and transfected with ECM830 electroporation system (BTX). Electroporation condition was performed by 1 pulse at 70 V discharge for 30 ms.

### Generation of CK2β-Cas9 stable cell lines

The CK2β CRISPR/Cas9 containing cas9, green fluorescent protein (GFP), gRNA expression and CK2β HDR expressing puromycin resistance, red fluorescent protein (RFP) Plasmids were purchased from Santa Cruz. HEK293 cells in a 6-well plate were co-transfected with 2 ug of CK2β CRISPR/Cas9 and 2 ug of CK2β HDR plasmids using Transporter™ 5 transfection reagent. The transfected HEK293 cells with GFP and RFP expression were selected with 5ug/ml puromycin (A1113803, Thermo Fisher Scientific) containing medium for 2 weeks. The puromycin‐resistant cells of CK2β stable knockout were maintained with 1 ug/ml puromycin, and confirmed by qRT-PCR with specific primers.

### NSC proliferation and differentiation assay

ReNcell CX cells (5 × 10^3^ cells /well) were seeded onto ultra-low attachment 12-well plates with ReN NSC maintenance medium. The medium was changed every 2 days, and the diameter of neurospheres was observed under a microscope. ReNcell CX cells were cultured in a differentiation medium [10 μM forskolin (F3917, Sigma-Aldrich), 20 ng/ml brain-derived neurotrophic factor (BDNF), 5 μM retinoic acid (R2625, Sigma-Aldrich) in NSC maintenance medium]. After 4 days, cells were fixed with 4% paraformaldehyde solution and immunostained with rabbit anti-MAP2 (Cell signalling Technology, Danvers, MA, US) and mouse anti-GFAP (Cell signalling Technology) antibodies.

### Conventional reverse transcription (RT)-PCR and quantitative (q)RT-PCR

Total RNA was isolated using an Rneasy mini kit (QIAGEN, Hilden, Germany). A 1 μg of total RNA was used for cDNA synthesis was performed using Verso cDNA Synthesis Kit (Thermo Fisher Scientific) according to the manufacturer’s instructions. For conventional RT-PCR, the cDNA was amplified with the following gene-specific primers. The PCR products were loaded on 1% agarose gel and photographed.

For qRT-PCR, qRT-PCR was performed with AriaMx Real-Time PCR System (Agilent, Santa Clara, CA, US). Reactions were prepared in a total volume of 14 µl containing: 2 µl of template, 0.6 µl of each primer (final concentration of 400 nM), 7 µl of 2 × SensiFAST SYBR No-ROX mix (Meridian bioscience Inc., OH, US). The PCR reaction was performed according to the manufacturer’s instructions, in brief, 95 °C for 3 min, followed by 40 cycles of 5 s at 95 °C and 10 s at 60 °C. Melting curve analysis was performed under the following condition: 30 s at 95 °C, 30 s at 65 °C, and 30 s at 95 °C. After normalization against GAPDH mRNA levels, relative quantification of gene expression was performed using the 2-ΔΔCT method. The primer sequences are provided in Additional file 2: Tables S4 and S5.

### Immunofluorescence analysis

Cells were fixed with 4% paraformaldehyde for 1 h at room temperature (RT) at around 20–22 ℃, followed by three washes in PBS and permeabilization with 0.1% Triton X-100 in PBS for 15 min at RT. After fixation, blocking was performed with blocking reagent (2% normal horse serum in PBS) for 1 h at RT. Primary antibodies were diluted to the indicated concentrations (Additional file 2: Table S1) in PBST (1 × PBS with 0.05% tween 20), incubated overnight at 4 °C, and washed three times in PBS. The secondary antibodies were incubated in PBST for 1 h at RT and washed three times in PBS. For nuclear counterstaining, DAPI solution (BD Biosciences, Franklin Lakes, NJ, US) was incubated for 5 min at RT in PBST, washed, mounted with vectorMount AQ mounting medium (H-5501, Vector Laboratories, Burlingame, CA, US), observed under a fluorescence microscope (IX71, Olympus, Tokyo, Japan), and verified the co-localization using CellSens software. All images were quantitated by ImageJ software.

For visualizing Endoplasmic reticulum (ER) in living cells, ER staining was performed according to the instructions of the manufacturer of ER-tracker kit (Thermo Fisher Scientific). Briefly, HeLa cells were incubated with 1 uM ER tracker dye in Hank's buffered salt solution (#14025092, HBSS, Thermo Fisher Scientific) for 30 min at 37 °C and the cells were fixed with 4% PFA in PBS for 2 min at 37 °C.

### Western blot analysis

Briefly, cells were lysed on ice using RIPA buffer [50 mM Tris–HCl, pH 7.5, 150 mM NaCl, 0.5 mM EDTA, 1% NP40, 0.1% SDS, 1 mM PMSF, 1 × protease inhibitor cocktail (Roche, Basel, Switzerland)], separated using 12% SDS-PAGE, and transferred to the PVDF membrane (Millipore). Membranes were incubated with specific primary antibodies in PBST overnight at 4 °C (Additional file 2: Table S1). Subsequently, the membrane was incubated with a secondary antibody in PBST containing 0.5% skim milk for 1 h at RT. The proteins were visualized using a chemiluminescence kit (Miracle-Star, Intron Biotech, Seoul, Republic of Korea).

### Protein stability analysis

HEK293 cells were transfected with 10 μg each of HA-SPAST-M1 or Flag-FBXL17 plasmids. After 24 h, cells were treated with 50 μg/ml cycloheximide for 0, 2, and 4 h. At the indicated time points, the cells were harvested, and proteins were detected using western blotting. The quantification experiment was performed three times under the same conditions. The signal intensity was determined using ImageJ software.

### Separation of cytosolic and nuclear extractions

Cells (3 × 10^6^ cells /100 mm dish) were transfected with 10 μg of plasmids as indicated and harvested 24 h post-transfection. A subcellular fraction from transfected cells was isolated using NE-PER Nuclear and Cytoplasmic Extraction Reagent kit (Thermo Fisher Scientific) according to the manufacturer’s instructions.

### Immunoprecipitation assays and pull-down assay

For the immunoprecipitation assay, HEK293 cells were transfected with 10 μg of plasmids as indicated and harvested 24 h after transfection. Cells were then lysed in NET gel buffer [50 mM Tris–HCl, pH 7.5, 150 mM NaCl, 0.1% NP-40, 1 mM EDTA, pH 8.0 containing protease inhibitor cocktails] by sonication, cleared by centrifugation at 13,000 rpm for 10 min at 4 °C and supernatants were incubated with bead-conjugated anti-Flag (Sigma-Aldrich) or anti-HA antibody (Thermo Fisher Scientific) for 3 h at 4 °C on a gentle rotator shaker. ReNcell CX (5 × 10^6^ cells) were lysed in NET gel buffer by sonication, centrifuged, and the supernatants were incubated with antibodies against FBXL17 or control IgG overnight at 4 °C. And then, the cell lysate containing antibodies was incubated with Protein A Magnetic Beads (Thermo Fisher Scientific) for 2 h at 4 °C. After incubation, the magnetic beads were placed into the magnet stand, washed three times with 1 ml PBST. Precipitated proteins were separated using SDS-PAGE and analyzed using western blotting.

For pull-down assay, cell lysates and reaction mixtures after in vitro assay were incubated with glutathione Sepharose 4B beads (Sigma-Aldrich, St Louis, MO, USA) or Ni–NTA agarose (QIAGEN) for 3 h at 4 °C with rotation. The beads were precleared three times in 1 ml PBST. After incubation, the bound proteins were washed three times in PBST and analyzed using western blotting with indicated antibodies.

### Purification of recombinant proteins expressed in* E. coli* and in vitro binding assay

*E. coli* BL21 cells containing the pET28a-His-SPAST plasmid were grown at 37 ℃ until the optical density at 600 nm (OD600) reached 1. Protein expression was induced by incubation with 0.5 mM isopropyl-b-D-thiogalactoside (IPTG, Sigma-Aldrich) and 2% Ethanol at 18 ℃ for 48 h. *E. coli.* BL21 cells containing pET28a-His-FBXL17-ΔNT1, pGEX4T1-GST-SPAST-M1, pGEX4T1-GST-KLHL, and pGEX4T1-GST plasmids were grown at 37 ℃ to reach an OD 600 nm of 0.8, induced with 1 mM IPTG and 2% ethanol at 20 ℃ for 24 h. The purification of his-tagged recombinant protein from induced *E. coli* was performed as previously described [1]. Briefly, cells were lysed using lysis buffer [20 mM Tris–HCl, 300 mM NaCl, 10 mM imidazole, and 1 mM Phenylmethanesulfonyl fluoride (PMSF, Roche), pH 7.5], incubated with Ni–NTA agarose for 3 h at 4 °C. The bead-protein complexes were loaded on a column and washed with washing buffer [20 mM Tris–HCl, 300 mM NaCl, and 20 mM imidazole, pH 7.5]. The washed beads were subsequently eluted in elution buffer [20 mM Tris–HCl and 250 mM imidazole, pH 7.5]. The eluted proteins were dialyzed in dialysis buffer [10 mM Tris–HCl, 10% glycerol, and 1 mM PMSF, pH 7.5] at 4 °C overnight. For purification of GST-tagged recombinant protein, cells were lysis with lysis buffer [1 × PBS containing 0.5% Triton X-100 and 1 mM PMSF] incubated with glutathione sepharose beads overnight at 4 °C. The bead-protein complexes were washed three times with washing buffer [1 × PBS containing 1 mM PMSF] and eluted in elution buffer [10 mM Tris–HCl and 150 mM NaCl, 25 mM reduced glutathione, pH 7.5]. The eluted proteins were dialyzed in a dialysis buffer [1 × PBS containing 10% glycerol, 1 mM PMSF].

For in vitro binding assay, purified recombinant proteins from *E. coli*, each 0.5 μg protein used per binding reaction was incubated in 200 μl binding buffer [1 × PBS, 0.1% NP-40 containing 1 mM PMSF and protease inhibitor cocktails], pulled-down using glutathione sepharose beads and analyzed using western blotting.

### In vivo and in vitro ubiquitination assay

In vivo and in vitro ubiquitination was performed as previously described [1, 2]. Briefly, for in vivo ubiquitination assay, HEK293 cells were transfected with 10 μg Flag-tagged SPAST-M1, Flag-SPAST-M87, the lysine to arginine mutants of SPAST-M1, and 5 μg HA-tagged ubiquitin plasmid using Transporter™ 5 transfection reagent. At 24 h after transfection, cells were treated 10 μM MG132 for 16 h, lysed in NET gel buffer, and the cell lysates were incubated using an anti-Flag magnetic bead overnight at 4 °C. The immunoprecipitated proteins were washed three times in PBST and analyzed using western blot using indicated antibodies. For in vitro ubiquitination assay, the reaction mixtures were incubated with 0.5 μg His-E1, 0.5 μg His-UbcH10b, 0.5 μg His-FBXL17-ΔNT1, 100 μg/reaction HeLa cell S100 extract, 5 μg GST-SPAST-M1, GST-KLHL or 5 μg GST, and 25 μg/mL Flag-ubiquitin (Boston Biochem, Cambridge, MA, USA) in reaction buffer [25 mM Tris–HCl, pH 7.5, 1 mM MgCl2, 2.5 mM DTT, 5 mM adenosine triphosphate containing the ATP-regeneration system [1 mM creatine phosphate, 1 mM creatine kinase, 0.5 μg/mL ubiquitin aldehyde] at 37 °C for 1.5 h, followed by pull-down using glutathione sepharose beads and analyzed using western blotting.

### In vitro casein kinase II (CK2) kinase assay

GST-tagged SPAST, KLHL, and GST alone or His-tagged FBXL17-ΔNT1 proteins were purified from the *E. coli* expression system. The recombinant proteins (each 0.5 μg/reaction) were incubated in kinase reaction buffer [50 mM Tris–Cl. pH7.5, 200 mM NaCl, 10 mM MgCl2, 2 mM EDTA, 1 mM DTT, 200 μM ATP] with 500 U casein kinase II (P6010, New England Biolabs, Ipswich, MA, US), composed of two α-subunits and two β-subunits in the presence or absence of 10 μCi of [γ-32P]ATP for 30 min at 30 ℃. The reaction was terminated by adding SDS sample loading buffer [50 mM Tris–Cl. pH 6.8, 2% SDS, 10% glycerol, 0.2% bromophenol blue dye, 4% β-mercaptoethanol]. After resolution using SDS-PAGE, the gel was transferred into the PVDF membrane and visualized by direct exposure of the membrane to X-ray film (AGFA, Belgium).

### Animal experiments and dissection of the forebrain and spinal cord from mouse embryos

Mice were housed in a specific-pathogen-free animal laboratory (humidity 60–65%; temperature 22 °C) in 12 h light–dark cycles. All animal housing and experiments conducted were in accordance with the Korea Research Institute of Bioscience and Biotechnology (KRIBB) Institutional Animal Care and Use Committee Guidelines (KRIBB-AEC-18090).

The mouse embryonic tissues were harvested for the day of vaginal plug was dated as embryonic day (E) 0.5. The dissection of spinal cords from embryos was performed as previously described [3, 4]. Briefly, the pregnant mouse (E 10.5–18.5) was anesthetized using carbon dioxide (CO_2_) gas as a euthanasia agent, dissected the lower abdomen, and the uteri containing embryos were placed in a 100-mm Petri dish containing ice-cold sterile HBSS. The extraembryonic membranes overlying the embryos were removed from cranial to caudal and separated from the forebrain to the spinal cord using a dissection microscope (Nikon C-PSN, Tokyo, Japan). The isolated tissues were washed in ice-cold PBS and analyzed using RT-PCR or western blotting.

### Mouse embryo tissue preparation and immunofluorescence assay

The pregnant mouse (E 14.5) was sacrificed by cervical dislocation and carefully removed the embryos from the uterus. Embryos were removed extraembryonic membranes and fixed overnight in 4% paraformaldehyde (PFA) in PBS at 4 °C following immersed in 30% sucrose in PBS for 48 h at 4 °C. For the frozen section, fixed embryos embedded in OCT compound (Tissue-Tek, #4583, Sakura Finetek USA, Torrance, CA, US), and sectioned at 10 μm thickness using a cryostat (CM1520, Leica, Wetzlar, Germany). The sections were treated with 0.5% Triton X-100 in PBS for 10 min and blocked with 2.5% normal horse serum in PBS for 1 h. After the blocking, sections were incubated with anti-SPAST antibody and anti-FBXL17 antibody for 24 h at 4 °C in PBS. After washing, the sections were proceeded according to the immunofluorescence assay method.

### Statistical analysis

The student’s *t*-test was used to determine statistical significance. Differences were considered statistically significant at *P* < 0.05.

## Results

### FBXL17 was a binding partner of SPAST and inversely correlated at the protein level in vitro and in vivo

**Fig. 1:**
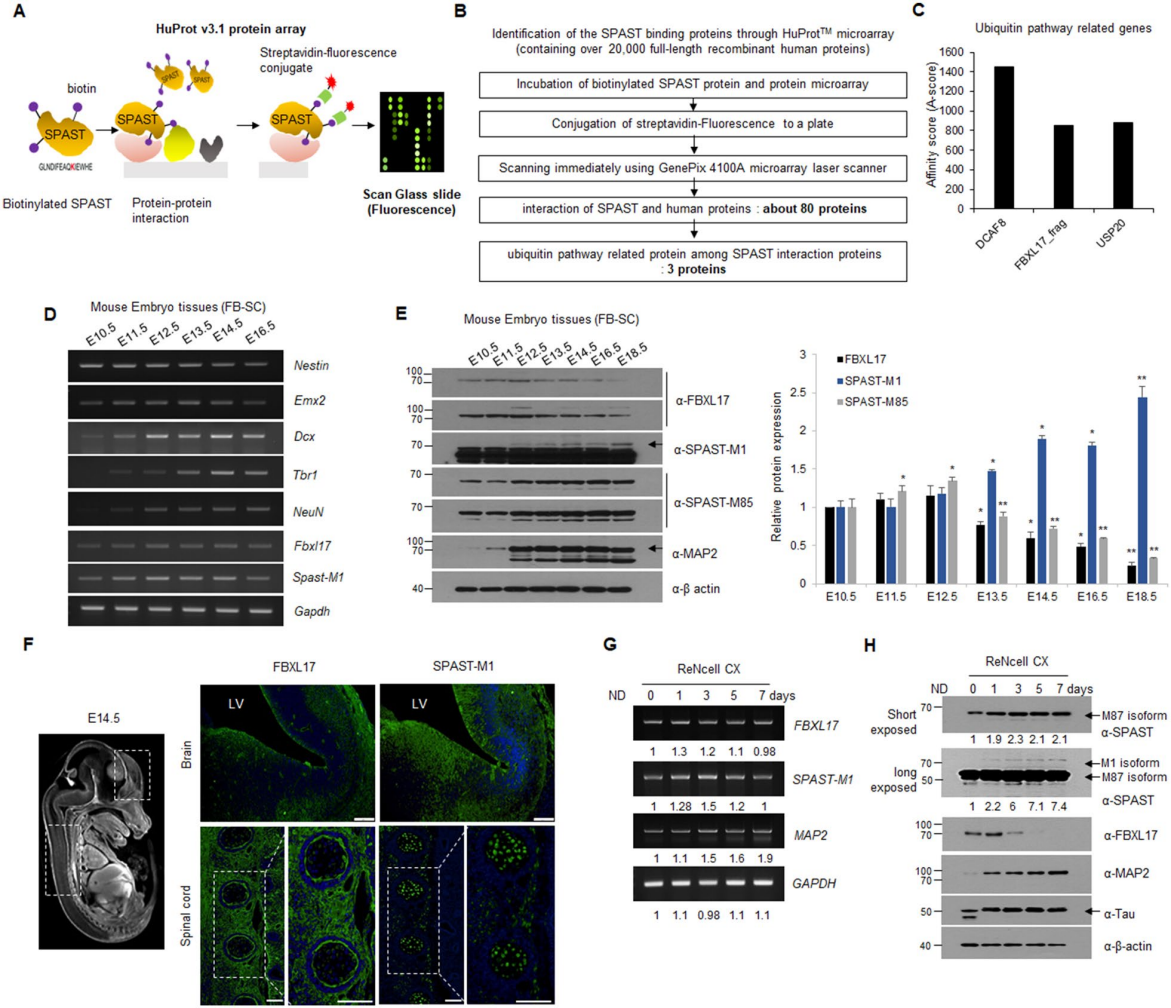
Screening of novel E3 ubiquitin ligase for SPAST using human protein microarray. **A** Schematic showing the screening of E3 ubiquitin ligase for SPAST-M1 through HuProt™ v3.1 protein microarray. **B** Schematic diagram of HuProt.™ protein microarray data analysis and filtering method involving SPAST protein. **C** Quantification of the relationship between SPAST and ubiquitin pathway-related genes using the array. **D** mRNA analysis of *Spast-M1*, *Fbxl17*, and neurogenesis marker genes from forebrain (FB) to spinal cord (SC) tissues of mouse embryos at indicated days. **E** Western blot analysis of SPAST-M1 or M85, FBXL17, and MAP2 as a neuronal marker from FB to SC tissues of mouse embryos at indicated days. Protein intensities quantified using densitometry and normalized to β actin expression. **F** Immunostaining of SPAST-M1 and FBXL17 on the brain and SC at the embryonic day 14.5. Scale bar: 100 μm. LV; lateral ventricular. **G** During neural differentiation, mRNA expression of *SPAST-M1* and *FBXL17* from ReNcell CX at the indicated days. **H** During neural differentiation, protein expression of SPAST-M1 or M87, FBXL17, MAP2, and Tau from ReNcell CX. The MAP2 and Tau expressions were used as neural markers. All experiments were performed in duplicate, data are expressed as the mean of two samples with standard deviation, and results are representative of two independent experiments. (*P < 0.05; ** *P* < 0.01)

Wild-type SPAST-M1 protein is more easily degraded by ubiquitin-dependent proteasomes than pathogenic mutants [10]. We hypothesized that enhancing the protein level of SPAST-M1 may mitigate the toxicity of mutants in HSP and screened the E3 ubiquitin ligase for SPAST-M1 protein. To identify the specific E3 ubiquitin ligase of SPAST-M1, protein–protein interaction screening was performed using biotinylated His-SPAST-M1 protein as a bait in a Huprot™ protein microarray containing over 20,000 full-length recombinant human proteins (Fig. 1A). The proteins interacting with SPAST-M1 were quantified using affinity (A) score through conjugation with streptavidin-fluorescence. As a result, a total of 80 proteins were identified, of which four associated with the ubiquitin pathway were selected (Fig. 1B) and quantified using the A-score (Fig. 1C). Three proteins involved in the ubiquitin–proteasome pathway were selected, namely DCAF8, FBXL17, and USP20. To confirm whether DCAF8 or FBXL17, as an E3 ubiquitin ligase, binds to SPAST-M1 after transfection with Flag-SPAST-M1 construct, HEK293 cells were immunoprecipitated using anti-Flag agarose beads and analyzed using western blot with antibodies against DCAF8 and FBXL17. We confirmed that SPAST-M1 directly binds only to FBXL17 (Additional file 1: Fig. S1). SPAST is tightly connected with HSP and plays a critical role in the neuronal development stage. SPAST is expressed in early-stage neural development and performs more specialized functions related to neuronal activities, such as axonal transport and the maintenance of excitatory synapses of motor neurons in the brain cortex and the spinal cord [14–16]. Therefore, we speculated that the precise regulation of SPAST by FBXL17 is critical for neuronal development and disease onset. To confirm SPAST and FBXL17 expression in the developing nervous system, we isolated mouse embryonic tissues, consisted of the brain and the spinal cord in mouse embryos at E10.5–18.5. RNA and protein were extracted from embryonic tissues and analyzed using RT-PCR or western blotting at the indicated embryonic days. The RNA levels of *Nestin*, *Emx2*, *Dcx*, *Tbr1*, and *NeuN* were used as neuronal development markers via RT-PCR analysis. The mRNA level of *Spast-M1* showed the greatest expression at E12.5, maintained up to E14.5, and rapidly decreased at E16.5. *Fbxl17* mRNA increased from E12.5 to E14.5 but decreased at E16.5, similar to *Spast-M1* (Fig. 1D and Additional file 1: Fig. S2). FBXL17 and SPAST-M85 proteins showed the greatest expression at E12.5, after which a continuous decline was confirmed. On the other hand, the protein expression of SPAST-M1 increased continuously from E13.5 to E18.5 (Fig. 1E). To confirm the reverse co-relation and localization of SPAST and FBXL17 expression, the brain and spinal cord were stained with specific SPAST-M1 and FBXL17 antibodies via immunofluorescence analysis at the embryonic day 14.5 (Fig. 1F). The reverse correlation of FBXL17 and SPAST-M1 expression is certified in the brain and spinal cord. After induction of neuronal differentiation in the neural progenitor cell line, ReNcell CX, mRNA expression of *SPAST-M1* and *FBXL17* was increased at the early time points after induction of neuronal differentiation (Fig. 1G). Under the same conditions shown in Fig. 1G, the FBXL17 protein level was decreased rapidly; conversely, SPAST-M1 protein gradually increased over time (Fig. 1H). Protein expression of SPAST and FBXL17 was inversely correlated in mouse embryonic tissues at the neural development stage, while ReNcell CX induced neuronal differentiation. This result confirmed that the SPAST-M1 protein is regulated by post-translational modification at the stage of neural development.

### SPAST-M1 protein interacted with SCF^FBXL17^ specifically via BTB domain existing M1, and not M87

**Fig. 2:**
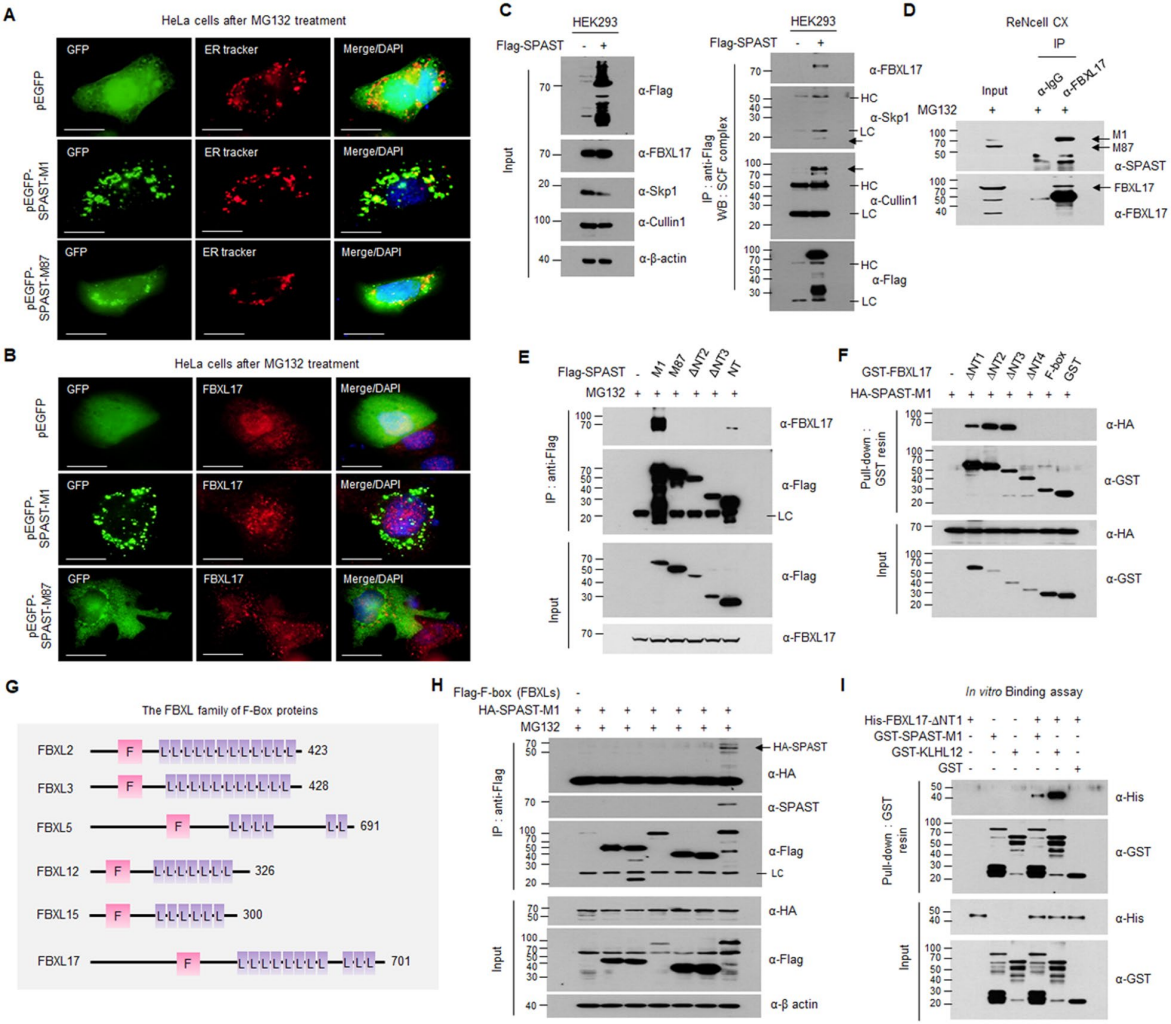
SPAST interacted with FBXL17, a substrate recognition component of SCF E3 ubiquitin ligase. **A** Immunostaining of GFP and ER in HeLa cells transfected with GFP empty, GFP-SPAST-M1, or M87 plasmids after MG132 treatment. DAPI was used for nuclear staining. Scale bar: 20 μm. **B** Immunostaining of GFP and endogenous FBXL17 in HeLa cells under the same conditions in (**A**) **C** Immunoprecipitation and western blot analysis of the interaction between Flag-tagged SPAST and endogenous SCF^FBXL17^ complex in HEK293 cells. **D** Interaction of endogenous SPAST and FBXL17 was analyzed by immunoprecipitation and western blotting from ReNcell CX. **E** Immunoprecipitation was performed using the anti-Flag magnetic bead in HEK293 cells transfected with the indicated plasmids, and IP samples were analyzed using western blotting. **F** The pull-down assay with glutathione agarose beads was performed in HEK293 cells transfected with the indicated plasmids and analyzed using western blotting. **G** Schematic showing the F-box domain (pink) and leucine-rich repeats domain (LRRs, purple) of the FBXL family members. **H** Immunoprecipitation and western blot analysis from HEK293 cells transfected with the indicated plasmids. **I** In vitro binding assay was performed with purified GST-tagged SPAST-M1, GST-KLHL, GST-alone, and His-tagged FBXL17-ΔNT1 (318–701 a.a.) from the *E.coli* expression system and analyzed using western blotting. All IP and WB experiments were performed in triplicate

Protein ubiquitination requires an enzymatic cascade containing the ubiquitin-activating enzyme E1, ubiquitin-conjugating enzyme E2, and ubiquitin ligase E3. FBXL17, which encodes a member of the F-box family of proteins, is a subunit of the SCF complex, consisting of Skp1, Cullin1, and F-box proteins as a function of E3 ubiquitin ligase, which recognizes substrates harboring the BTB domain and recruits these to the E3 ubiquitin ligase. Based on the interaction between SPAST-M1 and FBXL17 in the protein chip (Fig. 1C) and HEK293 cells (Additional file 1: Fig. S1B), we attempted to analyze the detailed domain of protein–protein interactions. First, we investigated the subcellular co-localization between the long isoform SPAST-M1 or short isoform SPAST-M87 to FBXL17 expression patterns in HeLa cells transfected with GFP-empty, GFP-SPAST-M1 or M87 plasmids, and treated with MG132 for inhibition of the ubiquitin–proteasome pathway. As shown in Fig. 2A, the M1 isoform with the hydrophobic region was highly localized to the endoplasmic reticulum (ER) membrane of the cytoplasmic and nuclear compartments. The M87 isoform is soluble and was more widely expressed in both the nucleus and cytoplasm than the M1 isoform, and some localized to the ER. For confirm the co-localization of SPAST and FBXL17, we stained with an anti-FBXL17 antibody and showed that endogenous FBXL17 was mainly expressed in the nucleus (Fig. 2B). Based on the above results, it was confirmed that SPAST M1 was localized to the ER and nucleus and co-localized with FBXL17 in the nuclear envelope and nuclear compartment (Fig. 2A, B). We next determined whether SPAST interacted with the SCF complex as an E3 ubiquitin ligase containing Skp1, Cullin1, and FBXL17. In HEK293 cells, SPAST interacted with the SCF complex, including FBXL17 (Fig. 2C). Because protein overexpression can affect protein interaction, we further comfirmed the interaction between endogenous SPAST-M1 and FBXL17 was confirmed in ReNcell CX (Fig. 2D). To determine which SPAST regions are required for interaction with FBXL17, we generated several N-terminal SPAST-truncated mutants into the pFlag-Tag2B vector (Additional file 1: Fig. S3A) and performed immunoprecipitation-western blot analysis using the indicated antibodies in HEK293 cells (Fig. 2E). FBXL17 interacted strongly with both SPAST-M1 (1–616 a.a.) and SPAST-NT (1–250 a.a.). In contrast, the M87 isoform of SPAST did not interact with FBXL17. It has been previously shown that FBXL17, as a substrate recognition component of the SCF complex, interacts with BTB proteins, and the BTB domain is required for the substrate-binding region of SCF^FBXL17^ [17]. Using the NCBI Multiple Sequence Alignment Viewer (Version 1.18.1), we found that the SPAST N-terminal region (50–120 a.a.) is highly conserved with BTB domain sequences of the identified BTB proteins (Additional file 1: Fig. S3B). These results demonstrate the interaction between FBXL17 and SPAST N-terminal region (50–86 a.a.) containing the predicted BTB domain. To determine which regions of FBXL17 are required for interaction with SPAST-M1, we examined HA-SPAST-M1 interaction with several N-terminal FBXL17 truncated mutants using a GST pull-down assay. The N-terminal truncated mutants of FBXL17 were generated from the F-box to the leucine-rich repeat (LRR) domain of substrate recognition by FBXL proteins (Additional file 1: Fig. S3C). The result shown that SPAST interacted strongly at 471–571 a.a region of FBXL17 (Fig. 2F). To investigate binding specificity between SPAST and FBXL17, we screened six human F-box/LRR-repeat proteins associated with brain and neural development. HEK293 cells were co-transfected with Flag-tagged F-box and HA-SPAST-M1 constructs and analyzed via immunoprecipitation-western blot analysis. We found that SPAST interacted with only the FBXL17 protein (Fig. 2G, H). To confirm the direct interaction between SPAST and FBXL17, recombinant GST-tagged SPAST-M1, GST-KLHL12, and His-tagged FBXL17-ΔNT1 (318-701a.a.) proteins were purified in an *E. coli* expression system, and the purity and identity of these proteins were confirmed using Coomassie Blue staining (Additional file 1: Fig. S6A). We then performed an in vitro binding assay and confirmed that His-FBXL17-ΔNT1 directly interacted with GST-SPAST-M1 and GST-KLHL12, but not GST alone (Fig. 2I). The GST-KLHL12 protein was identified as a substrate of FBXL17 and was used as a positive control in this study. Collectively, FBXL17 specifically interacted with SPAST-M1 via a leucine-rich substrate recognition site and BTB domain.

### The SCF^FBXL17^ E3 ubiquitin ligase complex degraded SPAST-M1 protein in the nuclear fraction in a proteasome-dependent manner

**Fig. 3:**
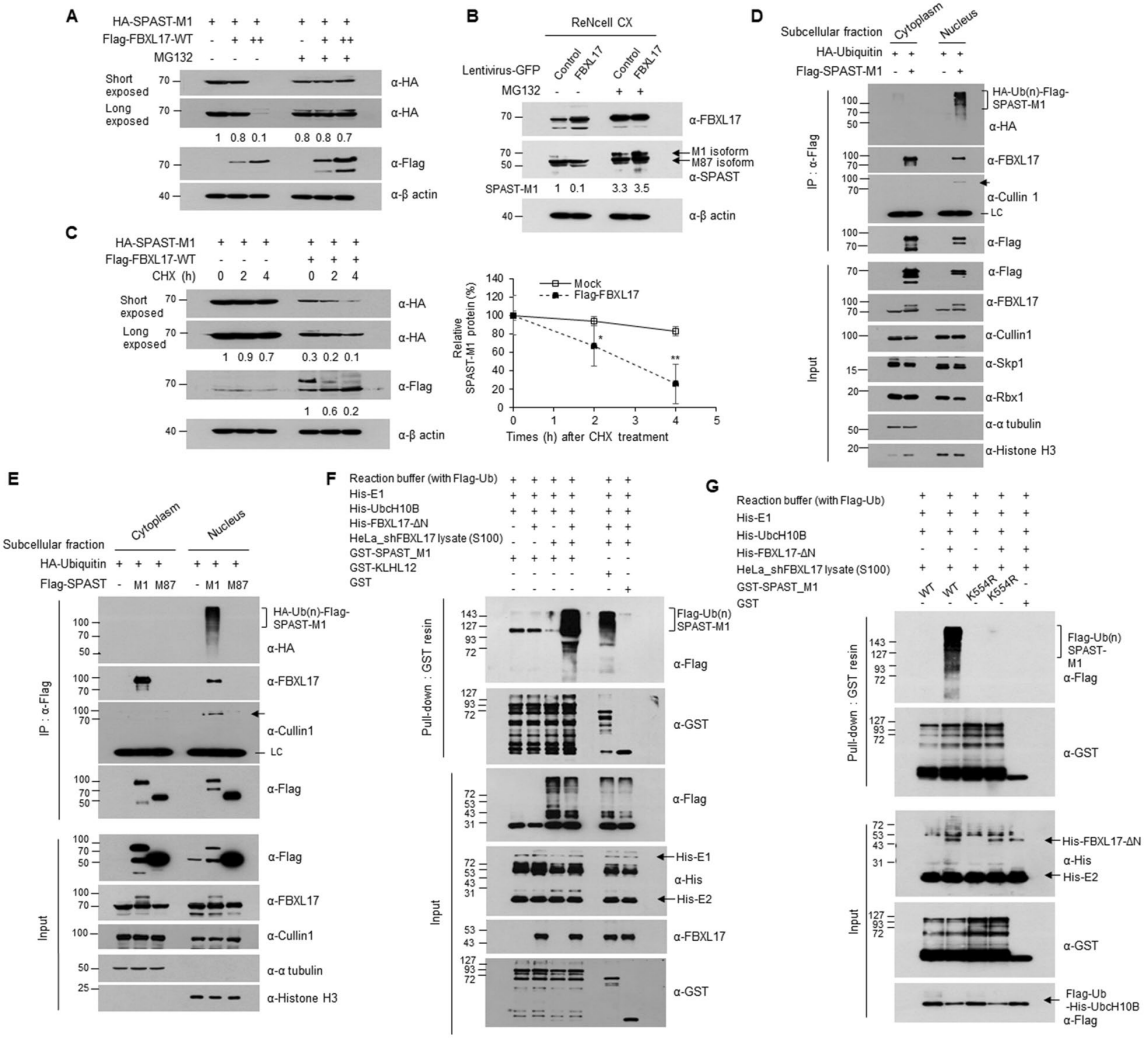
The SCF^FBXL17^ E3 ligase complex induces poly-ubiquitination and proteasomal degradation of SPAST. **A** HEK293 cells were transfected with indicated plasmids followed by 10 μM MG132 treatment for 16 h and analyzed using western blotting. **B** After transduction on ReNcell CX with a lentiviral-vector expressing FBXL17, protein expression was detected using western blotting in the presence or absence with MG132. **C** HEK293 cells were transfected with HA-tagged SPAST-M1 and Flag-tagged FBXL17 plasmids followed by 50 μg/ml cycloheximide (CHX) treatment for indicated times, analyzed using western blotting, and protein intensities quantified using ImageJ software. The assays were performed in triplicates and error bar represents SD. (*P < 0.05; ** *P* < 0.01). **D** HEK293 cells were transfected with indicated plasmids followed by MG132, and total cell lysates were separated into nuclear and cytoplasmic fractions. The same amounts of cell lysates were immunoprecipitated with anti-Flag-magnetic bead followed by western blotting with indicated antibodies. **E** HEK293 cells were transfected with Flag-SPAST-M1 or M87 plasmids, treated with MG132, and separated into subcellular fractions. Poly-ubiquitination of SPAST-M1 or M87 was analyzed using immunoprecipitation and western blotting. **F** In vitro ubiquitination assays were performed by incubating indicated proteins with the ATP-regeneration buffer system at 37 ℃ for 1.5 h. Proteins were purified from the *E. coli* expression system. The cytosolic S100 extract was prepared from HeLa cells transduced with Lenti-FBXL17 shRNA for 72 h. **G** In vitro ubiquitination assays were performed with GST-SPAST-M1-WT or K554R protein under the same conditions in (**F**), analyzed by western blotting. All IP and WB experiments were performed in triplicate, and results are representative of three independent experiments

Since the interaction with SCF^FBXL17^ led to proteasome-dependent degradation of the substrate, we speculated that poly-ubiquitination occurred on the SPAST-M1 protein only, not M87. To confirm whether the SPAST-M1 protein underwent ubiquitin-dependent degradation through the SCF^FBXL17^ complex, we co-transfected HA-SPAST-M1 and Flag-FBXL17 into HEK293 cells treated with or without MG132 for 16 h and performed western blotting and RT-PCR. As a result, Flag-FBXL17 expression dose-dependently downregulated the SPAST-M1 protein, and MG132 treatment effectively inhibited the decrease in SPAST-M1 protein. Still, it did not alter *SPAST-M1* mRNA via *FBXL17* expression (Fig. 3A and Additional file 1: Fig. S4A). In ReNcell CX, the protein of endogenous SPAST-M1 was remarkably decreased after lentivirus-mediated FBXL17 gene transduction, but not SPAST-M87 (Fig. 3B). To investigate the stability of SPAST protein in the presence and absence of FBXL17 expression, we treated HEK293 cells expressing HA-SPAST-M1 and Flag-FBXL17 with a de novo protein synthesis inhibitor, cycloheximide (CHX), for the indicated periods, then performed western blotting, and quantified the band intensity of SPAST-M1 protein using ImageJ software. As shown in Fig. 3C, the half-life of SPAST-M1 was more than 4 h in the absence of FBXL17, whereas the half-life of SPAST-M1 was approximately 3 h in the presence of FBXL17. Under the same conditions shown in Fig. 3C, the mRNA level of *SPAST-M1* did not significantly change in the presence or absence of *FBXL17* (Additional file 1: Fig. S4B). These results indicate that FBXL17-mediated post-translational modifications regulate SPAST. We found that GFP-tagged SPAST and endogenous FBXL17 were exclusively co-localized in the nucleus (Fig. 2B). To determine whether SPAST ubiquitination occurs via FBXL17 in the cytoplasm or nucleus, we transfected the indicated constructs into HEK293 cells, separated pure cytoplasmic and nuclear fractions, and performed an in vivo ubiquitination assay. SPAST-M1 interacted with FBXL17 in both the cytosolic and nuclear fractions. In contrast, SPAST-M1 interacted with Cullin1, a component of the SCF complex through FBXL17 in the nucleus, and the poly-ubiquitinated form of SPAST-M1 was only observed in the nuclear fraction (Fig. 3D). SPAST-M87 did not interact with FBXL17; therefore, the poly-ubiquitinated form of SPAST-M87 was not observed in any subcellular compartments (Fig. 3E). We used α-tubulin and histone H3 as markers for subcellular fractionation and the BDM-PUB server (http://bdmpub.biocuckoo.org/) to identify the lysine residues responsible for SPAST ubiquitination. The nine lysine residues, scored with medium and high confidence as predicted ubiquitination sites (Additional file 2: Table S6), and the interspecies functional conservation was confirmed using multiple alignment software (Clustal 2.1, Additional file 1: Fig. S5A, marked in red boxes). We generated SPAST mutants with lysine to arginine and performed an in vivo ubiquitination assay in HEK293 cells expressing the lysine-mutated SPAST protein. Mutation of lysine 554 into arginine strongly reduced the poly-ubiquitinated form of SPAST protein in the nuclear fraction (Additional file 1: Fig. S5B). To determine whether the SCF^FBXL17^ complex affected the ubiquitination of SPAST-M1, an in vitro ubiquitination assay was performed with E1 (His-E1), E2 (His-UbcH10b), F-box protein of the SCF complex (His-FBXL17-ΔNT1), and cytosolic extracts (S100) for SCF complex components as an E3 enzyme. The E1 and E2 proteins were purified from the *E. coli* expression system. The purity of these proteins was confirmed using Coomassie Blue staining (Additional file 1: Fig. S6A), and E1 and E2 enzyme activity was observed verified using an in vitro thiol ester assay (Additional file 1: Fig. S6B). The cytoplasmic S100 extracts were generated via hypotonic lysis from HeLa cells transduced with lentivirus-FBXL17 shRNA, while FBXL17 knockdown was confirmed using western blotting with the indicated antibodies (Additional file 1: Fig. S6C). SPAST was directly poly-ubiquitinated by the SCF^FBXL17^ complex as an E3 ubiquitin ligase in vitro (Fig. 3F). But, the arginine mutation of K554 of SPAST as major ubiquitination site was not observed poly-ubiquitination in vitro (Fig. 3G and Additional file 1: Fig. S6D). This result indicated that SPAST M1 is poly-ubiquitinated and degraded by the SCF^FBXL17^ complex.

### Phosphorylation was a critical mark for the ubiquitination and degradation of spastin protein

We observed that the ubiquitination of SPAST-M1 occurred only in the nucleus, with size differences observed between the nuclear and cytoplasmic forms of SPAST. The appearance of a larger SPAST-M1 in the cytoplasm led us to investigate the post-translational modification. In particular, casein kinase 2 (CK2) is known as a nominator for regulation and interaction with SPAST [18]. Thus, we hypothesized that SPAST-M1 was regulated by post-translational modification (PTM).

**Fig. 4:**
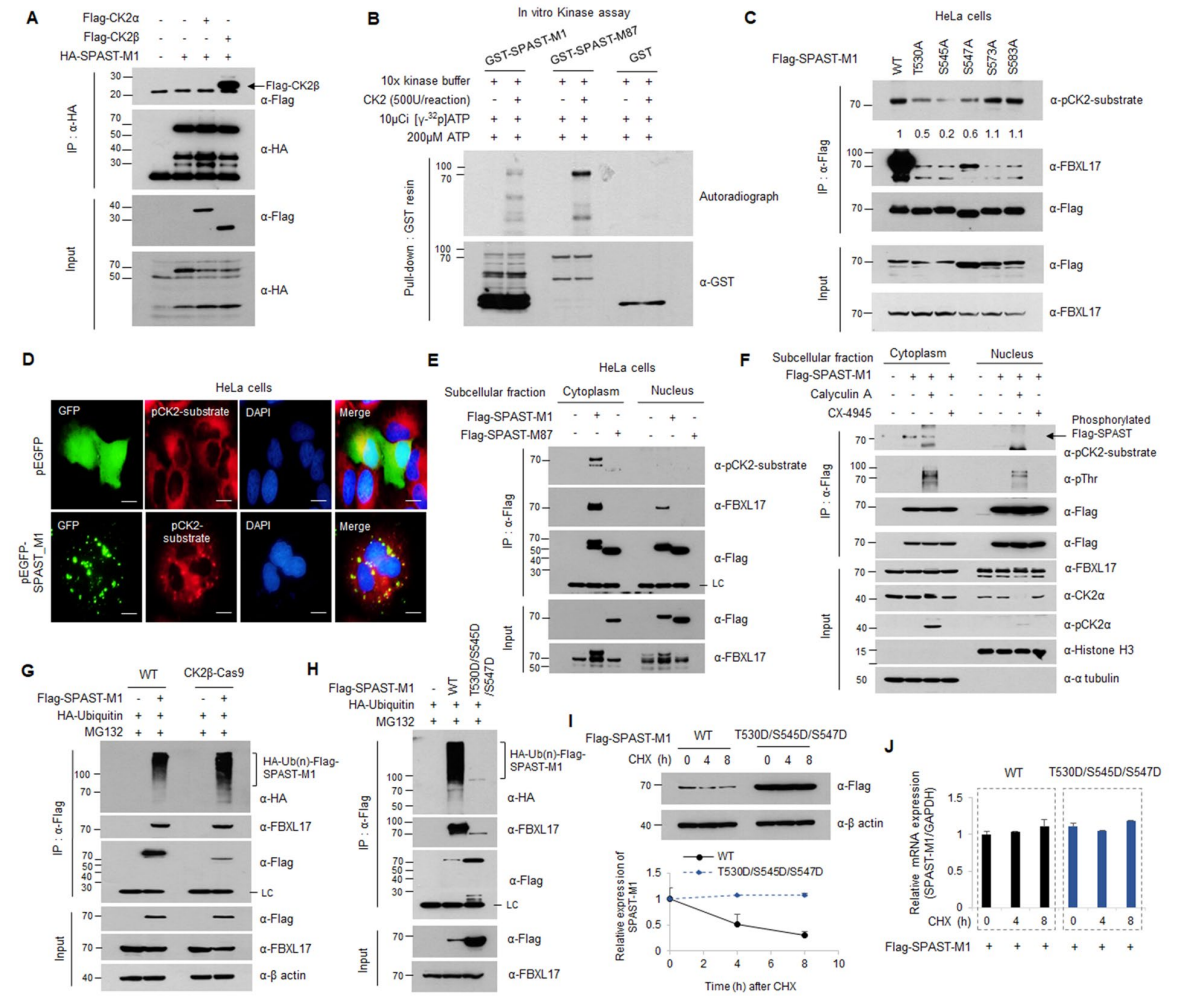
Ubiquitination of SPAST is regulated by CK2-dependent phosphorylation. **A** Interaction of CK2α, or Ck2β and SPAST in HEK293 cells transfected with indicated plasmids. **B** For In vitro kinase assay, the indicated proteins were purified from *E. coli* and incubated with recombinant CK2 protein in the presence of [γ^32^P]ATP. Autoradiography shows phosphorylated GST-SPAST. **C** Phosphorylation of the serine/threonine to alanine mutants of SPAST was analyzed using IP-western blotting in HeLa cells. **D** Immunostaining of GFP and phospho-CK2 substrate in HeLa cells transfected with GFP empty or GFP-SPAST-M1 plasmids. Scale bar: 50 μm. **E** After transfection of Flag-SPAST-M1 or M87 plasmids in HeLa cells, phosphorylation of SPAST-M1 or M87 was analyzed by IP-western blotting from nuclear and cytoplasmic fractions of HeLa cells. **F** HEK293 cells were transfected with indicated plasmids and treated with CX-4945 (for 24 h with 5uM) or calyculin A (for 15 min with 10 nM), followed by isolation of nuclear and cytoplasmic fractions and analysis using western blotting. **G** The poly-ubiquitination of SPAST-M1 was analyzed by IP-western blotting in the HEK293 WT or CK2β-Cas9 stable cell lines. **H** The poly-ubiquitination of SPAST WT or phosphomimetic mutant (triple aspartic acid substitution mutations; T530D, S545D, and S547D) was analyzed by IP-western blotting in the HEK293 cells. **I** The protein stability of WT and phosphomimetic mutant of SPAST was analyzed by western blotting after CHX treatment. **J** The mRNA level of WT and phosphomimetic mutant of SPAST under the same conditions as 4I. All experiments were performed in triplicate, and results are representative of three independent experiments

To confirm the PTM of SPAST-M1, we co-transfected HEK293 cells with expression plasmids and performed immunoprecipitation assays and western blotting and showed that SPAST-M1 only interacted with CK2β, but not with CK2α (Fig. 4A). Using an in vitro CK2 kinase assay, we confirmed that GST-tagged SPAST proteins containing the two main isoforms were phosphorylated by CK2 but not GST alone (Fig. 4B). Following the in vitro kinase assay, equal amounts of protein were confirmed using Coomassie Blue staining (Additional file 1: Fig. S7A). We found that the predicted serine/threonine sites of SPAST, including the CK2 consensus phosphorylation site [S/T–X–X–D/E] identified using Group-based prediction system 5.0 software (http://gps.biocuckoo.cn/, Additional file 2: Table S7). We confirmed that the predicted S/T sites are conserved in the three species (Additional file 1: Fig. S5A, marked in blue boxes). To determine the predicted S/T sites for CK2-mediated SPAST-M1 phosphorylation, we mutated these residues from serine/threonine to alanine (T530A, S545A, S547A, S573A, and S583A of SPAST), and the phosphorylation of these mutants was analyzed using immunoprecipitation-western blotting with an antibody against the CK2 phosphorylated consensus motif in HeLa cells. CK2-mediated phosphorylation of SPAST T530A, S545A, and S547A was reduced; thus, we confirmed that SPAST-M1 was phosphorylated by CK2 at multiple sites (Fig. 4C). To confirm whether CK2-mediated phosphorylation of SPAST occurs only in the cytoplasm fraction, HeLa cells were transfected with GFP-SPAST-M1 plasmid and analyzed using an immunofluorescence assay with a pCK2-substrate motif antibody. Immunofluorescence staining with pCK2-substrate motif antibody was markedly present in the cytosol of GFP-expressing HeLa cells and appeared strongly re-stained in the ER in the presence of GFP-SPAST-M1 (Fig. 4D). Both Flag-SPAST-M1 and M87 were detected by the anti-pCK2-substrate motif antibody only in the cytoplasmic fraction, consistent with the IF in Fig. 4D, E. However, only SPAST-M1 protein was detected at approximately 10 kDa greater form in the cytoplasm. The appearance of a significantly greater form suggested that multiple phosphorylation had occurred in SPAST-M1, but not SPAST-M87. Then, we examined multiple phosphorylations using an anti-pThr antibody in the presence of a CK2 regulator. CX4945 potently inhibited CK2 enzymatic activity, and calyculin A inhibited both PP1 and PP2A phosphatase activities. The protein phosphorylated by CK2 is dephosphorylated by PP2A; therefore, it is used as a CK2 inhibitor or activator. The SPAST-M1 protein was detected using an antibody against the CK2 phosphorylated consensus motif; SPAST-M1 phosphorylation was reduced following CX4945 treatment but increased following calyculin A treatment (Fig. 4F). To verify the significance of CK2β, we generated CK2β knockout cell line by the CRISPR/Cas9, and CK2β knockout was verified by qRT-PCR using specific CK2β primer (Additional file 1: Fig. S7B). And then, the change of the poly-ubiquitination of SPAST-M1 was confirmed by immunoprecipitation and western blotting. SPAST-M1 ubiquitination was slightly increased in CK2β-Cas9 cell line compared to the WT cell line, this result suggested that the poly-ubiquitination of SPAST was regulated by the CK2 phosphorylation-dependent manner (Fig. 4G). In addition, to confirm the crosstalk between phosphorylation and ubiquitination of SPAST-M1, we generated a phosphomimetic mutant of SPAST-M1 (triple aspartic acid substitution mutations; T530D, S545D, and S547D), and poly-ubiquitination of phosphomimetic mutant was analyzed by in vivo ubiquitination assay. As a result, the ubiquitination of the phosphomimetic mutant was not observed compared to WT (Fig. 4H). Correspondingly, the protein stability of the phosphomimetic mutants was dramatically increased after CHX treatment and there was no change at the RNA level compared to WT ( Fig. 4I, J).

In the above results, we showed that SPAST are a novel substrate of CK2 kinase. We was confirmed that the SPAST was phosphorylated by CK2 in the cytoplasm and only ubiquitinated by SCF^FBXL17^ complex in the nucleus. These results suggest that SPAST phosphorylation contributes to its protein stability.

### Specific inhibition of SCF^FBXL17^ E3 ubiquitin ligase results in reduced proteasome-dependent degradation of SPAST-M1

**Fig. 5:**
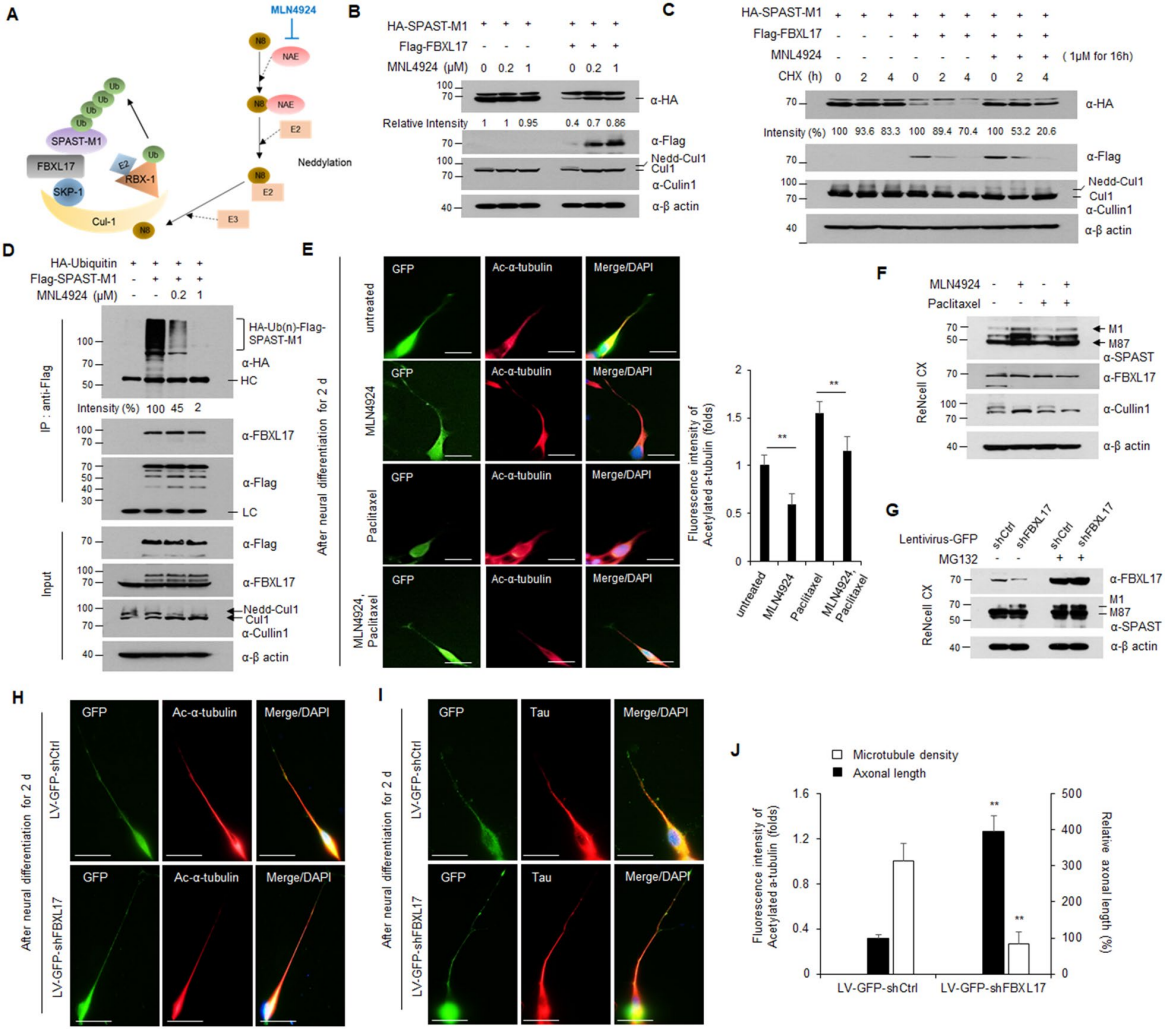
Inhibition of SCF.^FBXL17^ E3 ubiquitin ligase by MLN4924 stabilizes SPAST protein. **A** Schematic of mechanism for MLN4924-mediated inhibition of SCF complex. **B** The protein level of HA-SPAST-M1 in HEK293 cells treated with MLN4924 for 24 h in the presence or absence of FBXL17 expression. SPAST intensity bands were quantified using ImageJ software. **C** The half-life of HA-SPAST-M1 protein in HEK293 cells treated with 50 μg/ml CHX in the presence or absence of FBXL17 expression and/or MNL4924 treatment. SPAST intensity bands were quantified using ImageJ software, and the assays were performed in triplicates and error bar represents SD. **D** The poly-ubiquitination of HA-SPAST-M1 in HEK293 cells treated with MLN4924 at the indicated concentrations for 24 h. **E** ReNcell CX were transfected with a GFP-expressing vector, and incubated under neural differentiation condition for 2 d in the presence or absence of paclitaxel and MLN4924 singly and together. The cells were stained with GFP, and acetylated a-tubulin antibodies and quantified using ImageJ software. Scale bar: 20 μm. **F** Under the same condition as in Fig. 5E, protein expression of SPAST-M1, FBXL17, and Cullin1. **G** After transduction on ReNcell CX with a LV expressing shRNA for silencing of FBXL17, cells were analyzed using western blotting in the presence or absence with MG132. **H** and **I** ReNcell CX were transduced with lentiviral expressing FBXL17 shRNA (LV-GFP-shFBXL17) or scrambled non-targeting shRNA (LV-GFP-shCtrl), incubated under neuronal differentiation condition for 2 d, and immunostained with GFP, acetylated a-tubulin (as a marker for microtubule density), and Tau (as a marker for axonal length) antibodies. Scale bar: 20 μm. **J** Immunostaining was quantified using ImageJ software. All experiments were performed in duplicate, and results are representative of two independent experiments. (** *P* < 0.01)

Next, we determined whether SPAST-M1 protein levels increased via regulation of SCF^FBXL17^ E3 ubiquitin ligase activity. Cullin family members, a component of cullin-RING E3 ubiquitin ligase (CRL), require cullin neddylation for CRL activity [19]. MLN4924 is a neddylation inhibitor currently investigated in several phase 1–3 clinical trials on various malignancies (Fig. 5A) [20]. Thus, we investigated whether the reduction of SPAST-M1 protein by FBXL17 was inhibited after MLN4924 treatment. HEK293 cells were co-transfected with HA-SPAST-M1 or/and Flag-FBXL17 plasmids, treated with the indicated concentrations of MLN4924 for 24 h, lysed, and detected using western blot analysis. The decrease in SPAST-M1 protein by FBXL17 expression was restored by MLN4924 treatment in a dose-dependent manner (Fig. 5B). The efficacy of MLN4924 was confirmed by the reduction of neddylated Cullin1 following MLN4924 treatment. Under the same experimental conditions, no effect was observed on the mRNA levels of *SPAST-M1* by MLN4924 treatment (Additional file 1: Fig. S8A). To determine whether MLN4924 treatment prolonged the half-life of SPAST-M1, HEK293 cells with the indicated expression plasmids were treated with CHX for specified periods and analyzed using western blotting. When SPAST-M1 and FBXL17 are simultaneously expressed, the expression of SPAST-M1 was reduced to a greater extent (approximately 60% at the “0” time point) compared to the expression of only SPAST-M1. However, SPAST-M1 expression was restored by MLN4924 treatment in the presence of FBXL17 (Fig. 5C). The mRNA level of *SPAST-M1* was not changed under the same conditions as Fig. 5C (Additional file 1: Fig. S8B). To confirm whether inhibition of CRL E3 ligase activity affects SPAST ubiquitination, we performed an in vivo ubiquitination assay in the presence of the indicated concentrations of MLN4924. We showed that the poly-ubiquitinated form of SPAST-M1 was markedly reduced by MLN4924 treatment in a dose-dependent manner (Fig. 5D). Also, the endogenous SPAST-M1 expression was dose-dependently increased in ReNcell CX following MLN4924 treatment (Additional file 1: Fig. S9A). After neuronal differentiation in the presence or absence of MLN4924, we confirmed that microtubule stability was significantly reduced based on the increasing endogenous SPAST-M1 by MLN4924 treatment (Additional file 1: Fig. S9B). MLN4924 treatment increases SPAST protein levels in two different SPG4-HSP models, and MNL4924 restored para-physiological SPAST levels in the SPG4-HSP haploinsufficient context [21]. We investigated whether the increase in SPAST-M1 expression affected neural differentiation via MNL4924 treatment. Under neural differentiation conditions, paclitaxel-treated ReNcell CX inhibited axonal sprouting and significantly shortened the length of axons. MLN4924-treated cells increased axonal extension compared to the control cells. Also, ReNcell CX cells treated with paclitaxel and MLN4924 simultaneously restored axonal swelling and axonal length compared to cells treated with paclitaxel alone (Fig. 5E). MLN4924 treatment increased endogenous SPAST protein in ReNcell CX; however, the FBXL17 level was unaffected (Fig. 5F). Next, we generated lentiviral-mediated shRNA against FBXL17 or scramble shRNA in ReNcell CX. We confirmed that the endogenous SPAST-M1 protein increased by FBXL17 knockdown (Fig. 5G), furthermore, neurite extension induced compared to control cells (Fig. 5H–J). These results indicate that the enhancement of SPAST-M1 stability could be a therapeutic option to restore SPAST functionality.

### SPAST mutant, evading FBXL17-mediated regulation, is involved in HSP pathogenicity

**Fig. 6:**
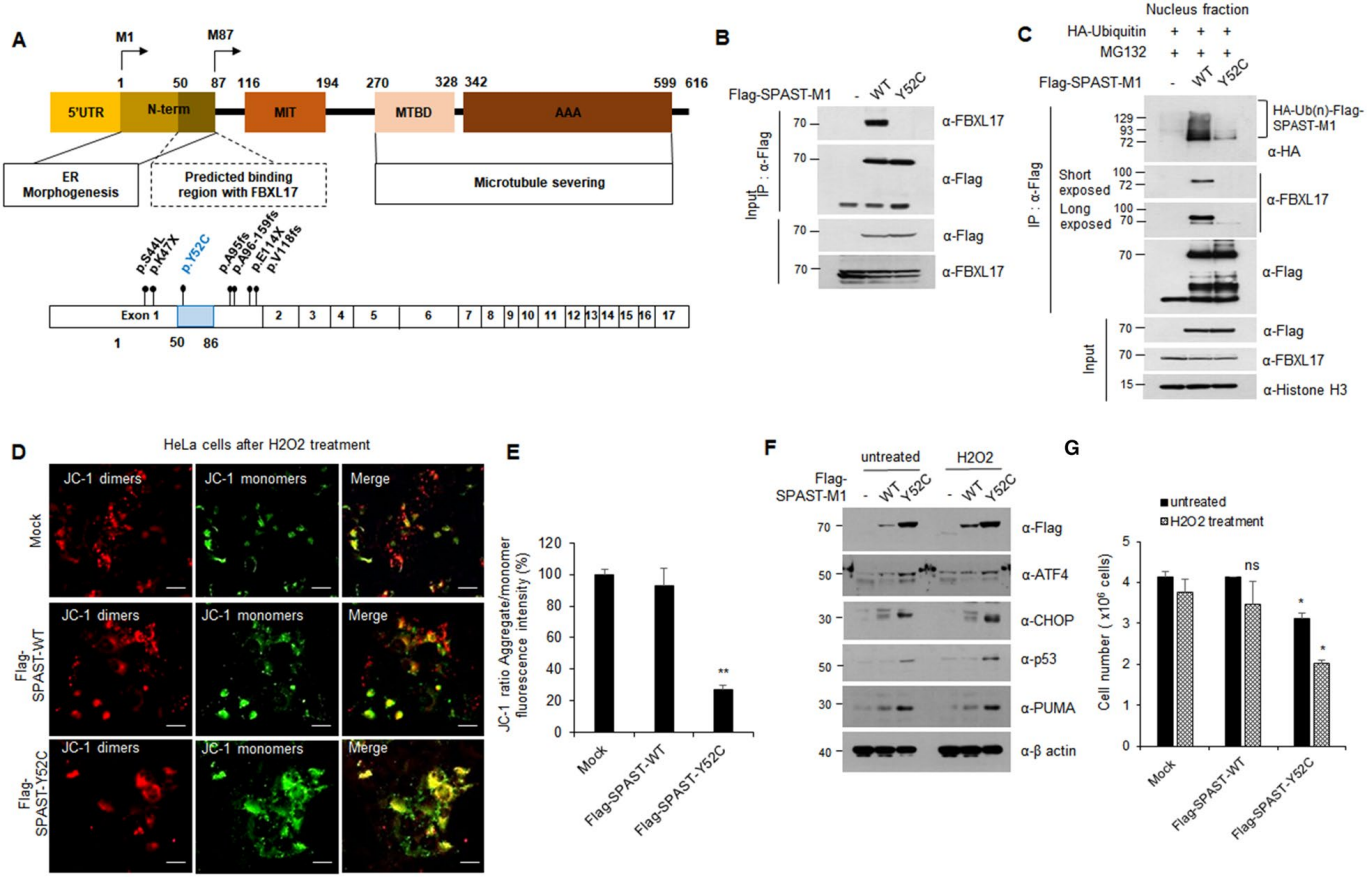
The pathogenicity of SPAST-Y52C mutation identified in a patient with HSP. **A** Structure of SPAST-M1 protein and its domains with the location of the mutations previously identified from patients with HSP. **B** The tyrosine 52 residue to cysteine mutant of SPAST (Y52C), identified from a Japanese patient with HSP, was constructed using primers to include the desired change. Interaction between SPAST WT or Y52C and FBXL17 in HEK293 cells transfected with indicated plasmids. **C** The poly-ubiquitination of WT and Y52C of Flag-SPAST-M1 from nucleus fraction of HEK293 cells transfected with indicated plasmids. **D** and **E** HeLa cells were transfected with Flag-SPAST-M1 WT or Y52C and treated with 100 μM H_2_O_2_ for 24 h. And then, cells were stained with JC-1 fluorescence dye and quantified with ImageJ software. (Scale bar: 50 μm). **F** Protein expressions of ER stress and apoptosis-related genes in HeLa cells transfected with WT or Y52C of Flag-SPAST-M1 in presence or absence of H2O2 treatment. **G** HeLa cells treated as in (**D**) was counted with an automated cell counter. All experiments were performed in duplicate, data are expressed as the mean of two samples with SD, and results are representative of two independent experiments. (*P < 0.05; ** *P* < 0.01; ns, not significant)

The interaction between SPAST-M1 and FBLX 17 is specific and plays a role in neuronal development and axonal extension. These results suggest that FBXL17-mediated SPAST deregulation induced abnormal SPAST protein quantity. Here, we searched SPAST pathogenic mutants harboring mutations on the binding region with FBXL17 (predicted BTB domain region; 50–120 a.a., Additional file 1: Fig. S3B) to show that this mechanism was implied in HSP pathogenicity. Eight SPAST mutants possessing a mutation in exon 1 were identified based on four references (Additional file 2: Table S8), and the position of mutations is shown in Fig. 6A, except for mutants p.Ala95Argfs, Term65 and p.A95fs [22–25]. Among these mutants, the SPAST Y52C mutant, first discovered in Japanese HSP patients, matched our hypothesis. Next, we examined the pathogenicity of the SPAST Y52C mutant based on the biochemical mechanism and cellular phenotypical changes. To investigate whether SPAST Y52C interacts with FBXL17, we performed immunoprecipitation-western blot analysis in HEK293 cells transfected with Flag-SPAST-WT or Y52C constructs (Fig. 6B). Moreover, the poly-ubiquitination of the SPAST Y52C mutant mediated by the interaction with FBXL17 as a substrate recognition component was confirmed (Fig. 6C). As shown above, in the SPAST Y52C mutant the FBXL17 binding site is destroyed, which increases the protein stability of the SPAST Y52C mutant via inhibition of poly-ubiquitination. Therefore, we confirmed that SPAST-M1 was poly-ubiquitinated in the nuclear compartment by binding to FBXL17 and SCF complex (Fig. 3). This finding, along with previous reports, led us to investigate other possibilities underlying pathogenicity, considering that oxidative stress plays a major role in the pathogenesis of several neurodegenerative diseases, including Alzheimer’s disease, Parkinson’s disease, amyotrophic lateral sclerosis, and hereditary spastic paraplegia [26, 27]. To determine the pathogenicity of the SPAST Y52C mutant, oxidative stress mediated apoptotic cell death as confirmed by the change in mitochondrial membrane potential evidenced by the JC-1 assay in HeLa cells transfected with Flag-SPAST WT or Y52C and quantified using ImageJ software (Fig. 6D, E). In addition, we performed western blot analysis in HEK293 cells transfected with Flag-SPAST WT or Y52C plasmids in the presence or absence of H_2_O_2_. Unlike SPAST WT expression, the expression of the mutant seemingly induced ER stress markers, such as ATF4 and CHOP, as well as apoptotic signaling, including p53 and PUMA-αβ, eventually leading to apoptotic cell death (Fig. 6F, G).

**Fig. 7:**
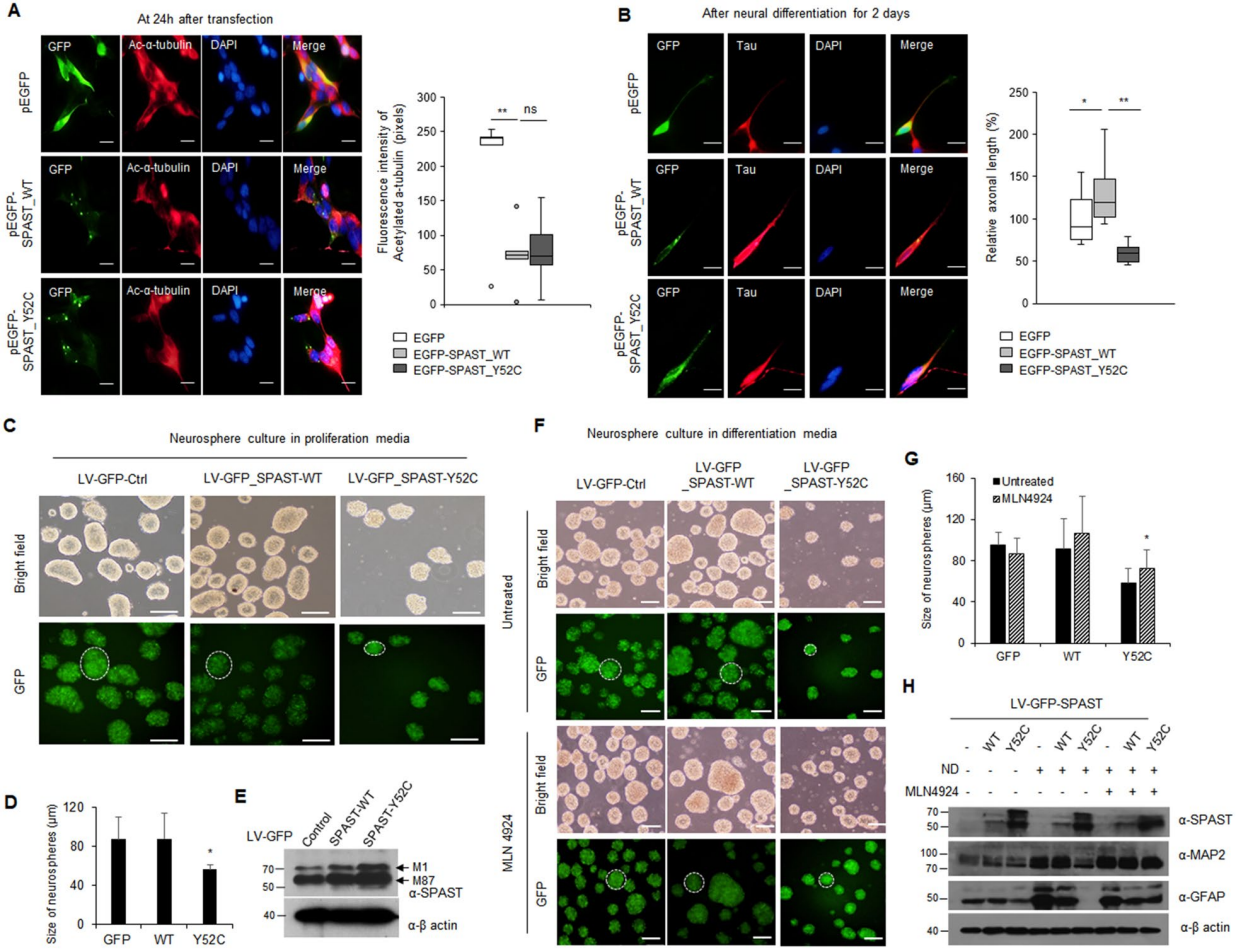
The pathogenicity of SPAST-Y52C was rescued by the inhibition of SCFFBXL17 in a 3D model. **A** Immunostaining of GFP and acetylated α-tubulin in ReNcell CX transfected with GFP-empty, WT and Y52C of GFP-SPAST-M1. Fluorescence intensities of acetylated α-tubulin quantified with ImageJ software. Scale bar: 50 μm. **B** After neuronal differentiation for 2 d, immunostaining of GFP and Tau in ReNcell CX transfected with GFP-empty, WT and Y52C of GFP-SPAST-M1. Scale bar: 20 μm. Quantification of mean axonal length in each group. **C** ReNcell CX were transduced with each indicated lentiviral vector (LV, with 5 MOI) and transferred to ultra-low binding well plates. Cells were cultured under orbital shaking conditions for 4 d, and observed under a microscope using bright field and GFP filter (Scale bar: 100 μm). **D** The neurospheres’ diameter was measured and quantified with ImageJ software. **E** The protein expression of SPAST-WT or Y52C under the same conditions in (**C**) **F** After transduction with each indicated lentiviral vector (5 MOI), ReNcell CX were cultured under orbital shaking for 4 d and then another 4 d under differentiation conditions with or without 5 μM MLN4924. Cells were observed under a microscope using bright field and a GFP filter (Scale bar: 100 μm). **G** The neurospheres’ diameter was measured and quantified with ImageJ software. **H** The protein expression of SPAST, MAP2 and GFAP under the same conditions in (**F**). MAP2 was used as a mature neural marker, and GFAP was used as an astrocyte marker. All experiments were performed in triplicate, data are expressed as the mean of three samples with SD, and results are representative of three independent experiments. (*P < 0.05; ** *P* < 0.01; ns, not significant)

Of note, mutations of SPAST causing HSP often impair the microtubule severing activity of SPAST. To evaluate the severing ability of the SPAST Y52C mutant, we transfected ReNcell CX with EGFP-empty, EGFP-SPAST-M1 WT, and Y52C plasmids by electroporation. After 24 h, we performed immunofluorescence analysis with acetylated α-tubulin antibodies and quantified fluorescence using the ImageJ software. Results indicated that SPAST Y52C expression significantly reduced acetylated α-tubulin levels similar to WT; thus, we confirmed that the SPAST Y52C mutation did not affect microtubule cleavage activity (Fig. 7A and Additional file 1: Fig. S10). To determine the difference in neuronal differentiation of the SPAST Y52C mutant, we performed immunofluorescence analysis with a Tau antibody (as an axonal marker) in ReNcell CX expressing EGFP, EGFP-SPAST WT, or Y52C incubated under neuronal differentiation conditions for 2 d. Axonal staining was measured and quantified using ImageJ software. Results showed that SPAST Y52C expression significantly reduced neurite length compared to SPAST WT (Fig. 7B). In addition, Since SPAST Y52C mutant affected cell death and axonal outgrowth, we aimed to further dissect the pathophysiological implications of a deregulated FBXL17-SPAST axis. ReNcell CX are neuronal progenitor cells that can grow fast and differentiate into multilineage cells of the brain, therefore they are an appropriate in vitro model to evaluate the pathogenicity of SPAST Y52C. Notably, providing 3D culture conditions help increase physiological relevance. To address this, ReNcell CX were transduced by SPAST WT or Y52C expressing lentiviral vector, proliferation and differentiation assays were performed in 3D conditions, and quantified by measuring the diameter. The size of neurosphere was significantly reduced in SPAST Y52C transduced cells compared to SPAST WT (Fig. 7C–E). Because the size of neurosphere was influenced by proliferation capacity, a reduced size implied that SPAST Y52C negatively regulate cell growth in 3D conditions. Furthermore, the capacity of multilineage differentiation was reduced by SPAST Y52C, but it was tangibly rescued by MLN4924 treatment used as an inhibitor of SCF^FBXL17^ ubiquitin ligase (Fig. 7F–H). Collectively, mutant proteins, which escapes regulation by the SCF^FBXL17^ E3 ubiquitin ligase complex, caused a loss of functionality with aberrant quantity and induced pathogenicity under oxidative stress via apoptotic signaling.

## Discussion

Herein, we discovered that the SPAST-M1 protein was precisely regulated by ubiquitination via the SCF^FBXL17^ complex. The ubiquitin–proteasome pathway plays a critical role in eliminating aberrant proteins in cells. The sequential activation of E1 (ubiquitin-activating enzyme), E2 (ubiquitin-transferring enzyme), and E3 (ubiquitin-ligating enzyme) induces the polyubiquitin chain on the lysine residue of the substrate and completes its degradation [13]. The SCF complexes are well-characterized, and the substrate specificity of SCF complexes is determined by the F-box protein, which harbors the F-box domain. More than 60 F-box proteins have been identified and target each substrate protein, thereby being implicated in controlling various diseases [11].

The protein expression patterns of SPAST-M1 were inversely correlated with FBXL17 during neuronal differentiation in mouse embryos and human neuronal progenitor cells (Fig. 1). While the transcriptional regulation of SPAST-M1 has been studied, nuclear respiratory factor-1 (NRF-1) and SRY-box transcription factor 11 (Sox11) play a role, the regulation of protein stability is not well understood [28]. Comparing the SPAST-M87 protein, low protein quantity SPAST-M1 protein was in neurons. In addition, SPAST itself is implicated in lysosome-mediated cellular compartment degradation via interaction with the endosomal sorting complex required for transport III (ESCRT-III), a component of the endocytosis machinery [29, 30] and plays a role in endocytosis and lysosomal protein degradation [31].

Specific interactions between SPAST-M1 and FBXL17 suggested the existence of a BTB domain in the N-terminus (aa 1–86) of SPAST-M1 (Fig. 2) since SCF^FBXL17^ eliminated nonfunctional proteins by interacting with FBXL17 and BTB domain proteins [17]. Indeed, searching the BTB domain using bioinformatics demonstrated that SPAST-M1 possesses a highly conserved BTB domain (Additional file 1: Fig. S3B). FBXL17 regulated the protein quantity of SPAST-M1 via nuclear poly-ubiquitination, while the absence of the BTB domain of SPAST-M87 maintained a greater protein quantity compared to SPAST-M1 (Fig. 3). SPAST-M1 was co-precipitated with Cullin1 and Skp1, compartments of the SCF complex, indicating that SPAST-M1 is a substrate SCF^FBXL17^. Few proteins, such as Sufu and BACH1, are substrates of SCF^FBXL17^, except for the BTB domain family [32, 33]. Here, we identified that SPAST-M1 is a new member of the SCF^FBXL17^ substrate.

Interestingly, interactions between SPAST-M1 and the SCF^FBXL17^ complex and poly-ubiquitination only occurred in the nucleus (Fig. 3D, E). Therefore, we investigated the differentiation of the modification status of SPAST-M1 protein in each cellular portion. F-box proteins require PTM of the substrate, such as phosphorylation and acetylation [13]. In particular, phosphorylation shows crosstalk with ubiquitination by regulating E3 ubiquitin ligase activity or binding affinity F-box protein [34]. We observed CK2-mediated phosphorylation of SPAST-M1 in vivo and in vitro. However, it was not critical for SPAST-M1 ubiquitination, while the phosphorylation of SPAST-M1 was required for evasion from the SCF^FBXL17^ complex (Fig. 4). SPAST-M1 harbors multiple phosphorylation sites, including CK2, since increasing protein size was relatively higher than ordinary phosphorylation, and the phosphorylated form was detected in the nucleus using an anti-pThr antibody. Previously, HIPK2 was discovered as a kinase and phosphorylated serine 268 of SPAST [21]. We identify that SPAST is a novel potential substrate for CK2 kinase protein, and phosphorylation of SPAST by CK2 contributes to its protein stabilization (Fig. 4G–I).

Regulation of CK2 may provide a method to restore functional SPAST-M1, but it seemed ineffective since SPAST-M1 was under control by multiple kinases; thus, the direct inhibition of poly-ubiquitination was tested. The Cullins family requires neddylation to activate E3 ubiquitin ligase for the substrate [35]. Therefore, we inactivated Cullins directly by inhibiting neddylation to increase SPAST-M1 protein levels and effectively induce neuronal differentiation (Fig. 5).

Among mutations occurring in exon 1of *SPAST* in the patient group, the Y52C mutation from the Japanese family was the only mutation in the BTB domain [22]. The Y52C mutant could not interact with FBXL17, thereby avoiding degradation by the SCF^FBXL17^ E3 ubiquitin ligase complex, resulting in abnormal accumulation of SPAST protein in the ER, which may induce pathogenic phenomena associated with oxidative stress in HSP. However, most *SPAST* mutations occur in the AAA ATPase region. Our findings provide no suggestions regarding therapeutic application against HSP induced by SPAST dysfunction due to mutations in this region but might provide a new insight for HSP therapeutics by regulating the quantity of SPAST-M1 protein.

## Conclusions

We discovered that the SCF^FBXL17^ E3 ubiquitin ligase complex recognizes SPAST-M1 via the BTB domain, further suggesting a mechanism to regulate poly-ubiquitination of SPAST-M1 for HSP therapeutics.

## Supplementary Information

**Additional file 1: Figure S1.** (A) Schematic image of the E3 ubiquitin ligase candidates of SPAST. (B) After transfection with the Flag-SPAST-M1 construct, HEK293 cells were immunoprecipitated, and analyzed using western blotting. **Figure S2.** The RT-PCR band density in Fig. 1D is generated using ImageJ software. **Figure S3.** (A) Schematic representation of SPAST N-terminal deletion mutants. Interaction capacity between SPAST deletion mutants and FBXL17 is indicated by an asterisk. (B) BTB domain sequences of the identified BTB proteins and SPAST-M1 were represented by multiple sequence alignments (C) Schematic representation of FBXL17 N-terminal deletion mutants. Interaction capacity between FBXL17 mutants and SPAST is indicated with the symbol. **Figure S4.** (A) mRNA expression was analyzed under the conditions shown in Fig. 3A and quantified using ImageJ software. (B) mRNA expression was analyzed under the conditions shown in Fig. 3C. Data are mean ± standard deviation. (ns, not significant). **Figure S5.** (A) Multiple sequence alignment of SPAST proteins in three species. The nine lysine residues or the predicted phosphorylation sites of SPAST confirmed its interspecies functional conservation using multiple alignment software (lysine residues, marked in red boxes; predicted S/T residues, marked in blue boxes). (B) For in vivo ubiquitination assay, HEK293 cells were transfected with indicated plasmids followed by MG132 treatment, and total cell lysates immunoprecipitated with anti-Flag-agarose gel followed by western blotting with indicated antibodies. **Figure S6.** (A) SDS-PAGE analysis of purified His-E1, His-UbcH10b, His-FBXL17-ΔNT1 (317-717a.a.), GST-SPAST-M1, GST-KLHL, and GST. All human recombinant proteins were expressed in *E. coli* and purified as described in the Materials and Methods section. Purified proteins were separated using SDS-PAGE gel and visualized using Coomassie Blue staining. (B) The E1/E2 thioester assay with His-E1, His-UbcH10b, and Flag-ubiquitin. (C) HeLa cells were transduced with scrambled shRNA control lentivirus or shRNA lentivirus targeted against FBXL17. After incubation for 72 h, S-100 cytosolic extract was prepared from the cells and analyzed via western blotting using antibodies against SCF^FBXL17^ components. (D) SDS-PAGE analysis of purified WT or K554R of GST-SPAST-M1 and GST proteins from the *E.coli* recombinant protein expression system. **Figure S7.** (A) In vitro CK2 kinase assay against SPAST. Proteins were separated using SDS-PAGE after the in vitro kinase assay in the presence or absence of CK2 and stained with Coomassie Blue staining demonstrating equal loading of the substrate. (B) mRNA level of CK2α and CK2β was analyzed by real-time qPCR in HEK293 WT or CK2β-Cas9 stable cell line (left). Data are represented as mean + /– standard deviation calculated from three replicates. The PCR products were visualized by 2% agarose gel electrophoresis (right). (**p < 0.01; ns, not significant). **Figure S8.** (A) mRNA expression was analyzed and quantified using RT-PCR under the conditions shown in Fig. 5B. (B) mRNA expression was analyzed using RT-PCR under the conditions shown in Fig. 5C and quantified. Data are mean ± standard deviation. (*p < 0.05; **p < 0.01; ns, not significant). **Figure S9.** (A) Rencell CX cells were treated with 0.2, 0.5 μM MLN4924 for 2 d, and analyzed using western blotting with the indicated antibodies. (B) After transfection with pEGFP plasmid, the cells were differentiated for 2 d in the presence or absence of 0.5 μM MLN4924 and stained with GFP and acetylated α-tubulin antibodies. Fluorescence intensities of acetylated α-tubulin quantified with ImageJ software. Data are mean ± standard deviation calculated from three replicates. Scale bar: 20 μm (*p < 0.05). **Figure S10.** (A) Immunostaining of GFP and Acetylated α-tubulin in HeLa cells transfected with GFP empty or GFP-SPAST-M1 plasmids, and (B) fluorescence intensities of acetylated α-tubulin quantified using ImageJ software. Data are mean ± standard deviation calculated from two replicates. Scale bar: 50 μm (**p < 0.01; ns, not significant).

**Additional file 2: Table S1.** Antibody list used in this study. **Table S2.** Reagents list used in this study. **Table S3.** Plasmid constructs used in the study. **Table S4.** The primer list for conventional RT-PCR. **Table S5.** The primer list for RT-qPCR. **Table S6.** The predicted ubiquitination sites of SPAST. **Table S7.** The predicted CK2 phosphorylation sites of SPAST. **Table S8.** SPAST exon 1 mutation and clinical summary found in HSP patients.

## Data Availability

Data will be made available on reasonable request.

## Abbreviations

MT: Microtubule
AAA: ATPase with diverse cellular activities
ER: Endosomal reticulum
AD: Autosomal dominant
HSP: Hereditary spastic paraplegias
FBXL17: F-box and leucine-rich repeat protein 17
SCF: Skp1-Cul1-F-box protein
FBXWs: F-box and WD repeats protein
FBXOs: F-box only protein
FBXLs: Leucine-rich repeats protein
BACH1: BTB and CNC homology 1
KLHL3: Kelch-like Family Member 3
KEAP1: Kelch-like ECH-associated protein 1
DCAF8: DDB1 And CUL4 Associated Factor 8
USP20: Ubiquitin Specific Peptidase 20
GST: Glutathione S-transferase
CK2: Casein kinase 2
CRL: Cullin-RING E3 ubiquitin ligase
NRF-1: Nuclear respiratory factor-1 a
Sox11: SRY-box transcription factor 11
ESCRT-III: Endosomal sorting complex required for transport III

## References

[1] Eckert T, Link S, Le DT, Sobczak JP, Gieseke A, Richter K (2012). Subunit interactions and cooperativity in the microtubule-severing AAA ATPase spastin. J Biol Chem.

[2] Solowska JM, Baas PW (2015). Hereditary spastic paraplegia SPG4: what is known and not known about the disease. Brain J Neurol.

[3] Tarrade A, Fassier C, Courageot S, Charvin D, Vitte J, Peris L (2006). A mutation of spastin is responsible for swellings and impairment of transport in a region of axon characterized by changes in microtubule composition. Hum Mol Genet.

[4] Fassier C, Tarrade A, Peris L, Courageot S, Mailly P, Dalard C (2013). Microtubule-targeting drugs rescue axonal swellings in cortical neurons from spastin knockout mice. Dis Model Mech.

[5] Allison R, Edgar JR, Pearson G, Rizo T, Newton T, Günther S (2017). Defects in ER–endosome contacts impact lysosome function in hereditary spastic paraplegia. J Cell Biol.

[6] Lo Giudice T, Lombardi F, Santorelli FM, Kawarai T, Orlacchio A (2014). Hereditary spastic paraplegia: clinical-genetic characteristics and evolving molecular mechanisms. Exp Neurol.

[7] Stevanin G, Ruberg M, Brice A (2008). Recent advances in the genetics of spastic paraplegias. Curr Neurol Neurosci Rep.

[8] Dion PA, Daoud H, Rouleau GA (2009). Genetics of motor neuron disorders: new insights into pathogenic mechanisms. Nat Rev Genet.

[9] Stone MC, Rao K, Gheres KW, Kim S, Tao J, La Rochelle C (2012). Normal spastin gene dosage is specifically required for axon regeneration. Cell Rep.

[10] Lim JH, Kang HM, Jung H-R, Kim D-S, Noh KH, Chang TK (2018). Missense mutation of SPAST protein (I344K) results in loss of ATPase activity and prolonged the half-life, implicated in autosomal dominant hereditary spastic paraplegia. Biochim Biophys Acta (BBA) Mol Basis Dis..

[11] Skaar JR, Pagan JK, Pagano M (2013). Mechanisms and function of substrate recruitment by F-box proteins. Nat Rev Mol Cell Biol.

[12] Huang X, Dixit VM (2016). Drugging the undruggables: exploring the ubiquitin system for drug development. Cell Res.

[13] Mason B, Laman H (2020). The FBXL family of F-box proteins: variations on a theme. Open Biol.

[14] Sherwood NT, Sun Q, Xue M, Zhang B, Zinn K (2004). Drosophila spastin regulates synaptic microtubule networks and is required for normal motor function. PLoS Biol.

[15] Plaud C, Joshi V, Marinello M, Pastré D, Galli T, Curmi P (2017). Spastin regulates VAMP7-containing vesicles trafficking in cortical neurons. Biochim Biophys Acta (BBA) Mol Basis Dis..

[16] Jeong B, Kim TH, Kim D-S, Shin W-H, Lee J-R, Kim N-S (2019). Spastin contributes to neural development through the regulation of microtubule dynamics in the primary cilia of neural stem cells. Neuroscience.

[17] Mena EL, Jevtić P, Greber BJ, Gee CL, Lew BG, Akopian D (2020). Structural basis for dimerization quality control. Nature.

[18] Leo L, Weissmann C, Burns M, Kang M, Song Y, Qiang L (2017). Mutant spastin proteins promote deficits in axonal transport through an isoform-specific mechanism involving casein kinase 2 activation. Hum Mol Genet.

[19] Enchev RI, Schulman BA, Peter M (2015). Protein neddylation: beyond cullin–RING ligases. Nat Rev Mol Cell Biol.

[20] Nawrocki ST, Griffin P, Kelly KR, Carew JS (2012). MLN4924: a novel first-in-class inhibitor of NEDD8-activating enzyme for cancer therapy. Expert Opin Investig Drugs.

[21] Sardina F, Pisciottani A, Ferrara M, Valente D, Casella M, Crescenzi M (2020). Spastin recovery in hereditary spastic paraplegia by preventing neddylation-dependent degradation. Life Sci Alliance..

[22] Ishiura H, Takahashi Y, Hayashi T, Saito K, Furuya H, Watanabe M (2014). Molecular epidemiology and clinical spectrum of hereditary spastic paraplegia in the Japanese population based on comprehensive mutational analyses. J Hum Genet.

[23] Kadnikova V, Rudenskaya G, Stepanova A, Sermyagina I, Ryzhkova O (2019). Mutational spectrum of Spast (Spg4) and Atl1 (Spg3a) genes in Russian patients with hereditary spastic paraplegia. Sci Rep.

[24] Lindsey J, Lusher M, McDermott C, White K, Reid E, Rubinsztein D (2000). Mutation analysis of the spastin gene (SPG4) in patients with hereditary spastic paraparesis. J Med Genet.

[25] Svenson IK, Ashley-Koch AE, Gaskell PC, Riney TJ, Cumming WK, Kingston HM (2001). Identification and expression analysis of spastin gene mutations in hereditary spastic paraplegia. Am J Hum Genet.

[26] Chiurchiù V, Orlacchio A, Maccarrone M. Is modulation of oxidative stress an answer? The state of the art of redox therapeutic actions in neurodegenerative diseases. Oxidative Med Cell Longevity. 2016;2016. 10.1155/2016/7909380 PMC4736210 26881039

[27] Wali G, Kumar KR, Liyanage E, Davis RL, Mackay-Sim A, Sue CM (2020). Mitochondrial function in hereditary spastic paraplegia: deficits in SPG7 but not SPAST patient-derived stem cells. Front Neurosci.

[28] Henson BJ, Zhu W, Hardaway K, Wetzel JL, Stefan M, Albers KM (2012). Transcriptional and post-transcriptional regulation of SPAST, the gene most frequently mutated in hereditary spastic paraplegia. PLoS ONE.

[29] Reid E, Connell J, Edwards TL, Duley S, Brown SE, Sanderson CM (2004). The hereditary spastic paraplegia protein spastin interacts with the ESCRT-III complex-associated endosomal protein CHMP1B. Hum Mol Genet.

[30] Allison R, Lumb JH, Fassier C, Connell JW, Ten Martin D, Seaman MN, et al. An ESCRT–spastin interaction promotes fission of recycling tubules from the endosome. J Cell Biol. 2013:jcb. 201211045. 10.1083/jcb.201211045 PMC3734076 23897888

[31] Lauwers E, Erpapazoglou Z, Haguenauer-Tsapis R, André B (2010). The ubiquitin code of yeast permease trafficking. Trends Cell Biol.

[32] Raducu M, Fung E, Serres S, Infante P, Barberis A, Fischer R (2016). SCF (Fbxl17) ubiquitylation of Sufu regulates Hedgehog signaling and medulloblastoma development. EMBO J.

[33] Tan M-KM, Lim H-J, Bennett EJ, Shi Y, Harper JW (2013). Parallel SCF adaptor capture proteomics reveals a role for SCFFBXL17 in NRF2 activation via BACH1 repressor turnover. Mol Cell.

[34] Hunter T (2007). The age of crosstalk: phosphorylation, ubiquitination, and beyond. Mol Cell.

[35] Zhou L, Jiang Y, Luo Q, Li L, Jia L (2019). Neddylation: a novel modulator of the tumor microenvironment. Mol Cancer.

