# Supplementary material for: FBXL17/spastin axis as a novel therapeutic target of hereditary spastic paraplegia
Source: Cell Biosci. 2022 Jul 22;12:110. doi: 10.1186/s13578-022-00851-1 (PMC9308218; doi:10.1186/s13578-022-00851-1)
Supplement: Supplementary file 1 — Additional file 1: Figure S1. (A) Schematic image of the E3 ubiquitin ligase candidates of SPAST. (B) After transfection with the Flag-SPAST-M1 construct, HEK293 cells were immunoprecipitated, and analyzed using western blotting. Figure S2. The RT-PCR band density in Fig. 1D is generated using ImageJ software. Figure S3. (A) Schematic representation of SPAST N-terminal deletion mutants. Interaction capacity between SPAST deletion mutants and FBXL17 is indicated by an asterisk. (B) BTB domain sequences of the identified BTB proteins and SPAST-M1 were represented by multiple sequence alignments (C) Schematic representation of FBXL17 N-terminal deletion mutants. Interaction capacity between FBXL17 mutants and SPAST is indicated with the symbol. Figure S4. (A) mRNA expression was analyzed under the conditions shown in Fig. 3A and quantified using ImageJ software. (B) mRNA expression was analyzed under the conditions shown in Fig. 3C. Data are mean ± standard deviation. (ns, not significant). Figure S5. (A) Multiple sequence alignment of SPAST proteins in three species. The nine lysine residues or the predicted phosphorylation sites of SPAST confirmed its interspecies functional conservation using multiple alignment software (lysine residues, marked in red boxes; predicted S/T residues, marked in blue boxes). (B) For in vivo ubiquitination assay, HEK293 cells were transfected with indicated plasmids followed by MG132 treatment, and total cell lysates immunoprecipitated with anti-Flag-agarose gel followed by western blotting with indicated antibodies. Figure S6. (A) SDS-PAGE analysis of purified His-E1, His-UbcH10b, His-FBXL17-ΔNT1 (317-717a.a.), GST-SPAST-M1, GST-KLHL, and GST. All human recombinant proteins were expressed in E. coli and purified as described in the Materials and Methods section. Purified proteins were separated using SDS-PAGE gel and visualized using Coomassie Blue staining. (B) The E1/E2 thioester assay with His-E1, His-UbcH10b, and Flag-ubiquitin. (C) [file 13578_2022_851_MOESM1_ESM.docx]

**FBXL17/spastin axis as a novel therapeutic target of hereditary spastic paraplegia**

Hyun Mi Kang^1^, Dae Hun Kim^1,2^, Mijin Kim^1,3^, Yoohong Min^1,4^, Bohyeon Jeong^1,2^, Kyung Hee Noh^1^, Da Yong Lee^1,2^, Hyun-Soo Cho^1,2^, Nam-Soon Kim^1,2^, Cho-Rok Jung^1,2*^ and Jung Hwa Lim^1*^

^1^ Korea Research Institute of Bioscience and Biotechnology (KRIBB), 125 Gwahak-ro, Daejeon, Republic of Korea.

^2^ Department of Functional Genomics, Korea University of Science and Technology (UST), 217 Gajeong-ro, Daejeon, Republic of Korea.

^3^ Department of Microbiology, Chungbuk National University, 28644, Chungbuk, Republic of Korea.

^4^ Department of Biology, Chungnam National University, 34134 Daejeon, Republic of Korea.

*Correspondence to Cho-Rok Jung ([crjung@kribb.re.kr](mailto:crjung@kribb.re.kr)) and Jung Hwa Lim ([jhwa@kribb.re.kr](mailto:jhwa@kribb.re.kr)), KRIBB, Daejeon 34141, Republic of Korea, Tel: 82 42 879 8175, Fax: 82 42 860 4597

**Supplementary figure legends**


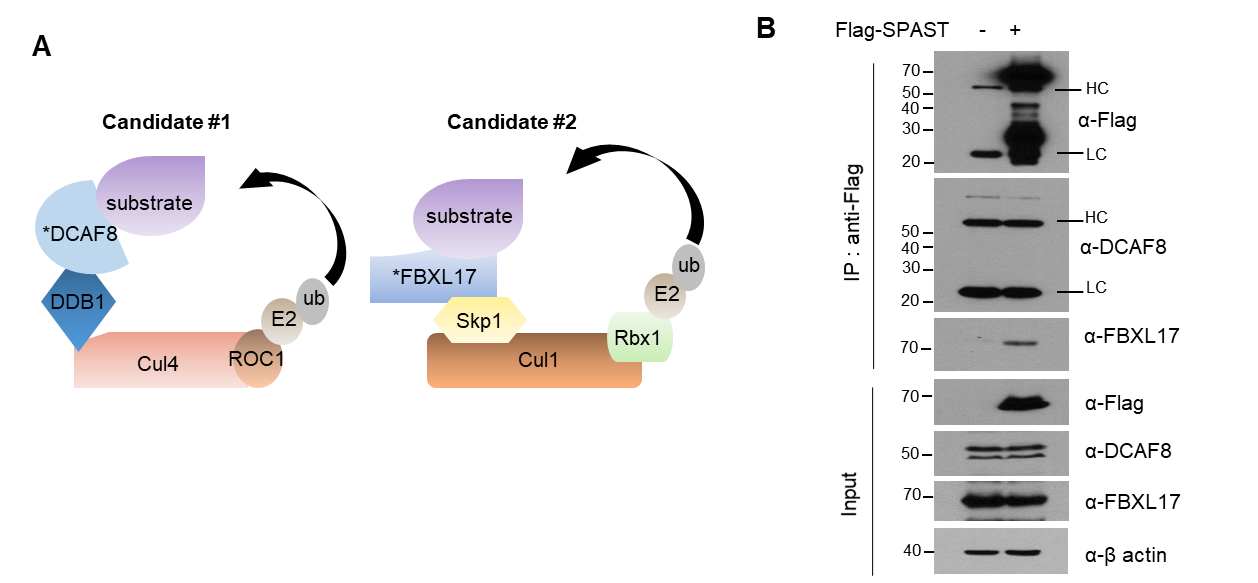


**Supplementary Figure 1.** (A) Schematic image of the E3 ubiquitin ligase candidates of SPAST. (B) After transfection with the Flag-SPAST-M1 construct, HEK293 cells were immunoprecipitated, and analyzed using western blotting.


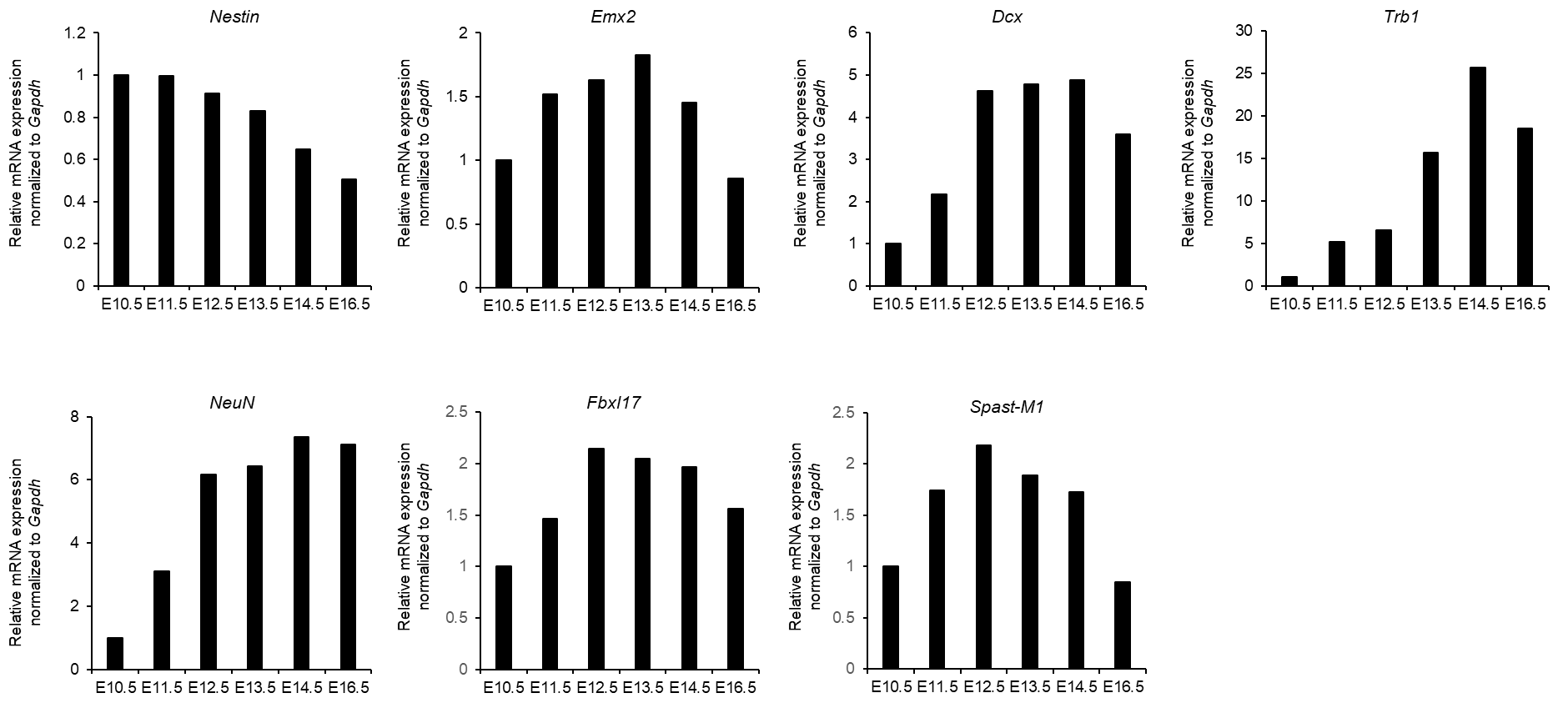


**Supplementary Figure 2.** The RT-PCR band density in Figure 1D is generated using ImageJ software.


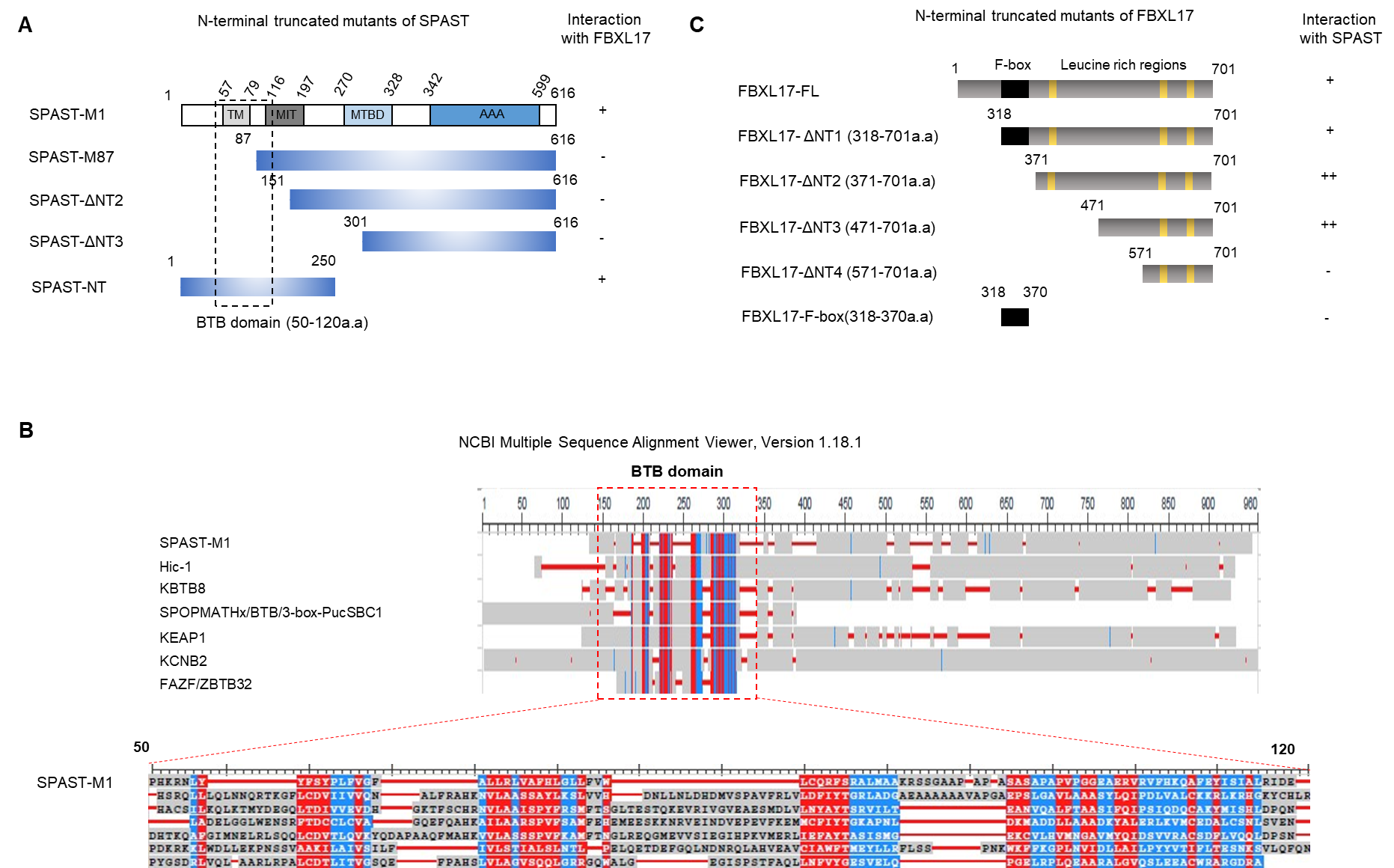


**Supplementary Figure 3.** (A) Schematic representation of SPAST N-terminal deletion mutants. Interaction capacity between SPAST deletion mutants and FBXL17 is indicated by an asterisk. (B) BTB domain sequences of the identified BTB proteins and SPAST-M1 were represented by multiple sequence alignments (C) Schematic representation of FBXL17 N-terminal deletion mutants. Interaction capacity between FBXL17 mutants and SPAST is indicated with the symbol.


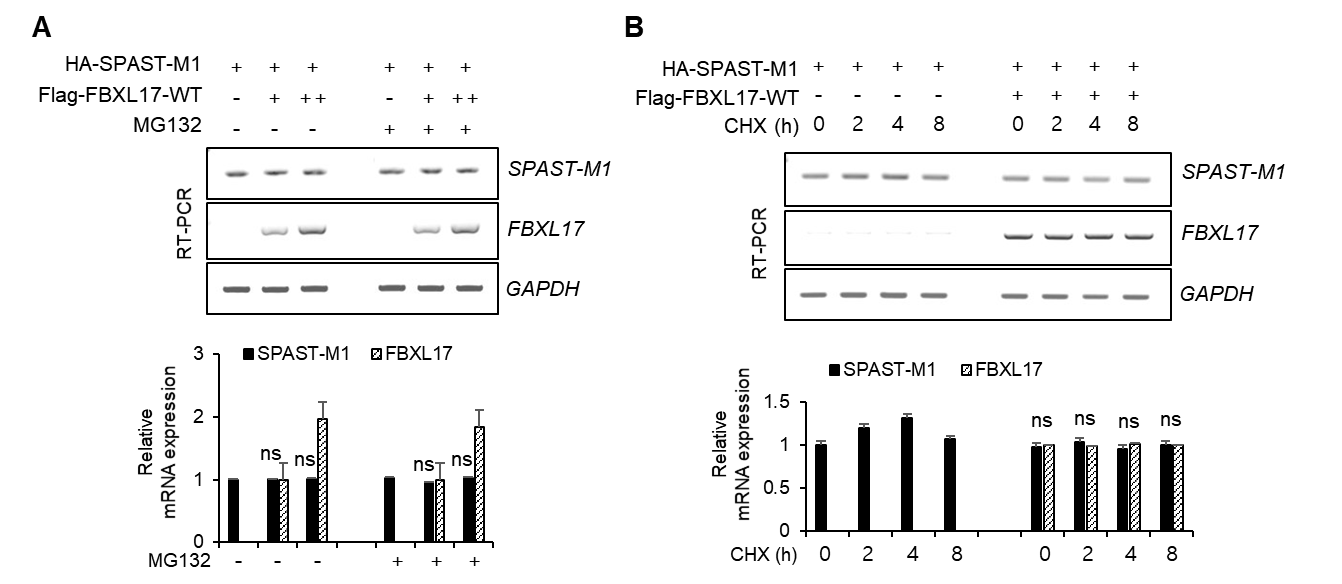


**Supplementary Figure 4.** (A) mRNA expression was analyzed under the conditions shown in Figure 3A and quantified using ImageJ software. (B) mRNA expression was analyzed under the conditions shown in Figure 3C. Data are mean ± standard deviation. (ns, not significant)


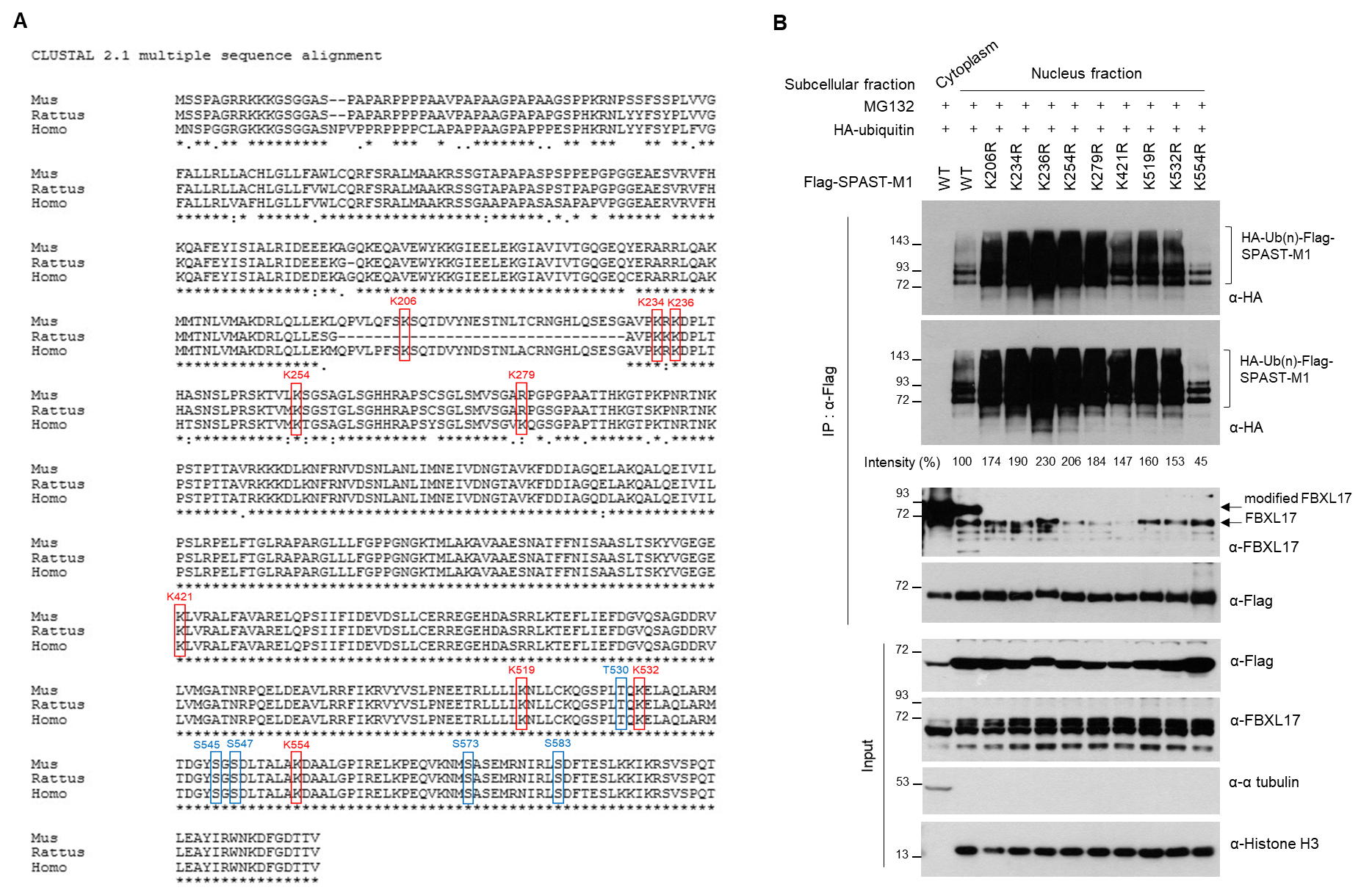


**Supplementary Figure 5.** (A) Multiple sequence alignment of SPAST proteins in three species. The nine lysine residues or the predicted phosphorylation sites of SPAST confirmed its interspecies functional conservation using multiple alignment software (lysine residues, marked in red boxes; predicted S/T residues, marked in blue boxes). (B) For in vivo ubiquitination assay, HEK293 cells were transfected with indicated plasmids followed by MG132 treatment, and total cell lysates immunoprecipitated with anti-Flag-agarose gel followed by western blotting with indicated antibodies.


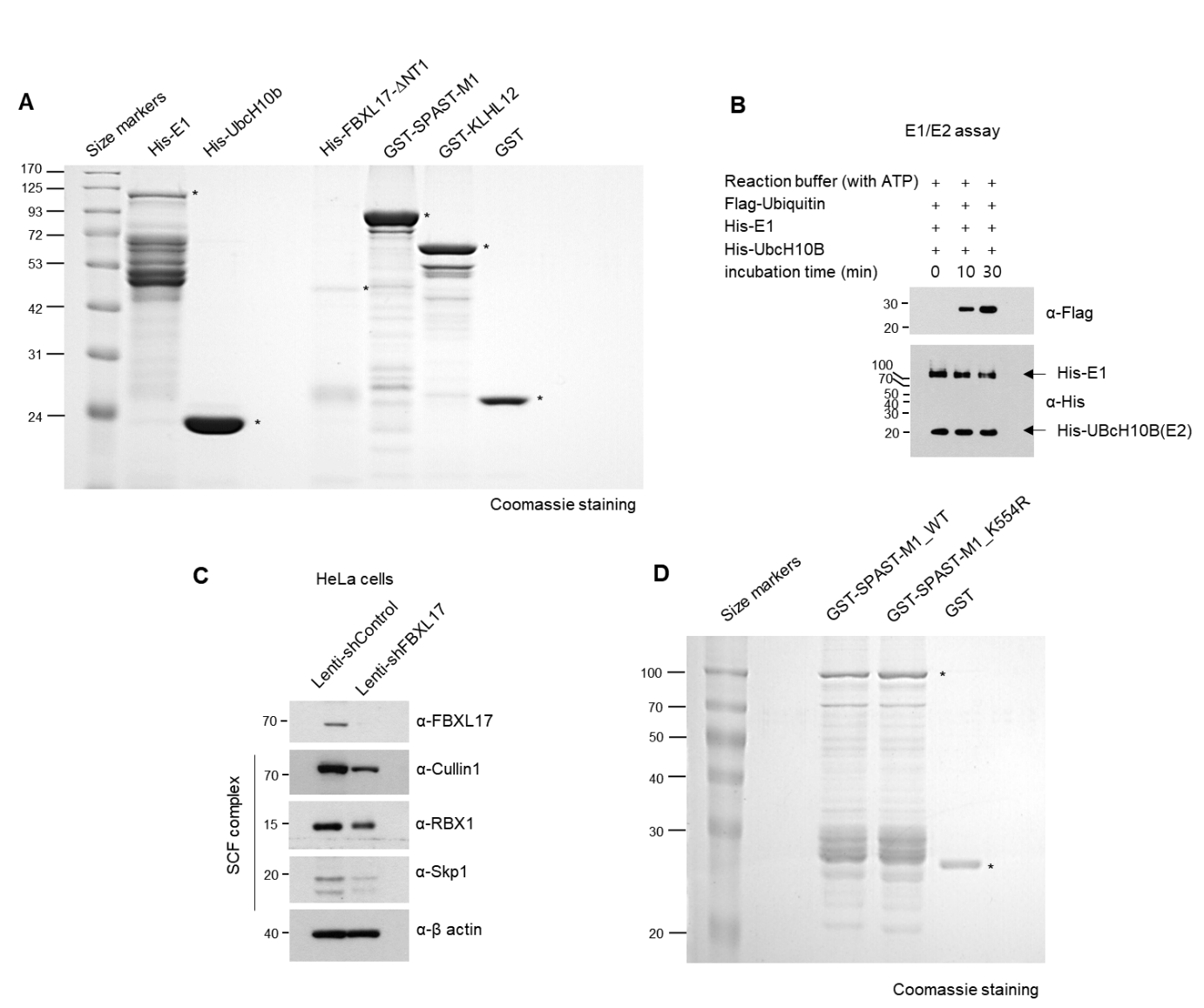


**Supplementary Figure 6.** (A) SDS-PAGE analysis of purified His-E1, His-UbcH10b, His-FBXL17-ΔNT1 (317-717a.a.), GST-SPAST-M1, GST-KLHL, and GST. All human recombinant proteins were expressed in *E. coli* and purified as described in the Materials and Methods section. Purified proteins were separated using SDS-PAGE gel and visualized using Coomassie Blue staining. (B) The E1/E2 thioester assay with His-E1, His-UbcH10b, and Flag-ubiquitin. (C) HeLa cells were transduced with scrambled shRNA control lentivirus or shRNA lentivirus targeted against FBXL17. After incubation for 72 h, S-100 cytosolic extract was prepared from the cells and analyzed via western blotting using antibodies against SCF^FBXL17^ components. (D) SDS-PAGE analysis of purified WT or K554R of GST-SPAST-M1 and GST proteins from the *E.coli* recombinant protein expression system.

**
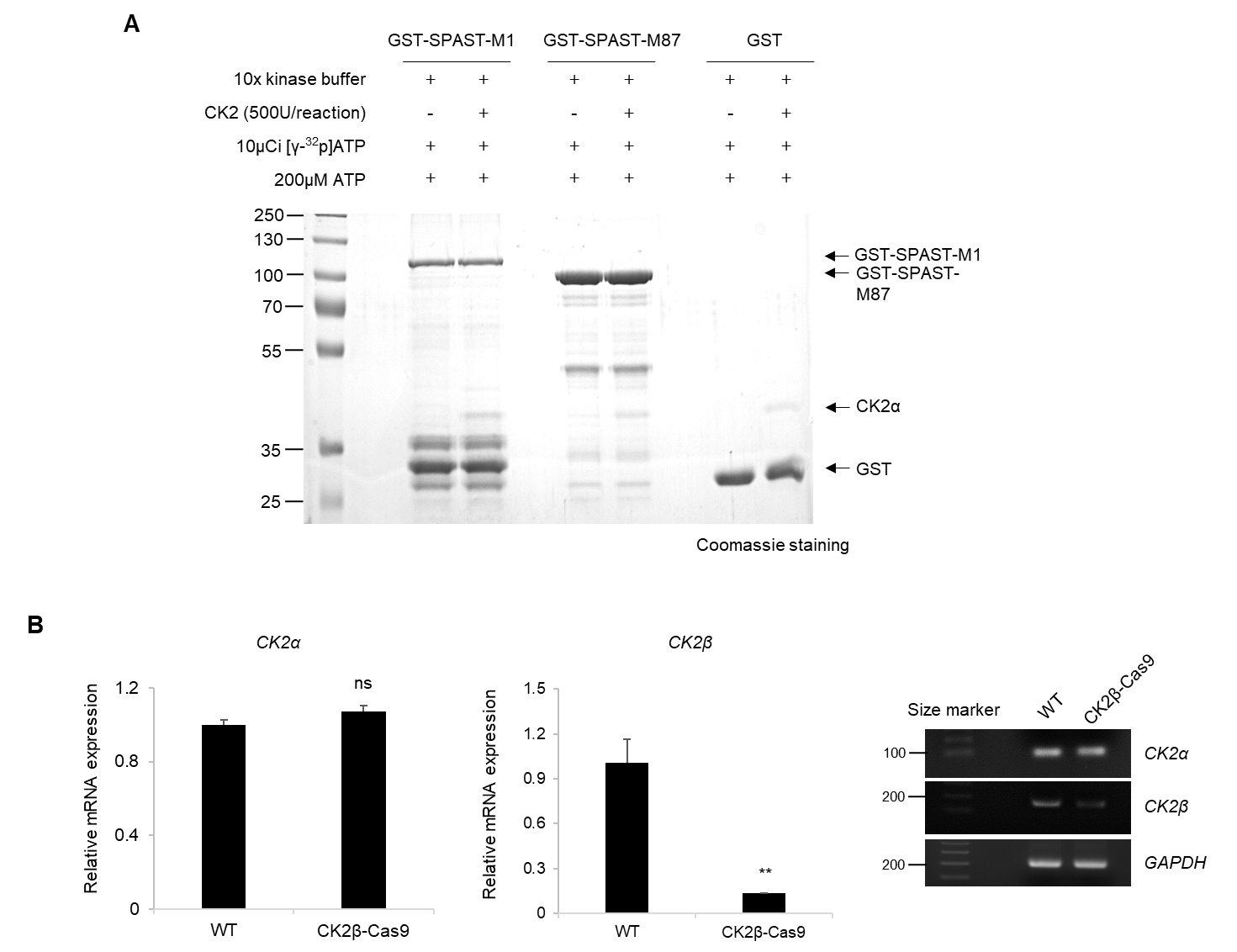
**

**Supplementary Figure 7.** (A) *In vitro* CK2 kinase assay against SPAST. Proteins were separated using SDS-PAGE after the *in vitro* kinase assay in the presence or absence of CK2 and stained with Coomassie Blue staining demonstrating equal loading of the substrate. (B) mRNA level of CK2α and CK2β was analyzed by real-time qPCR in HEK293 WT or CK2β-Cas9 stable cell line (left). Data are represented as mean +/– standard deviation calculated from three replicates. The PCR products were visualized by 2% agarose gel electrophoresis (right). (**p < 0.01; ns, not significant)


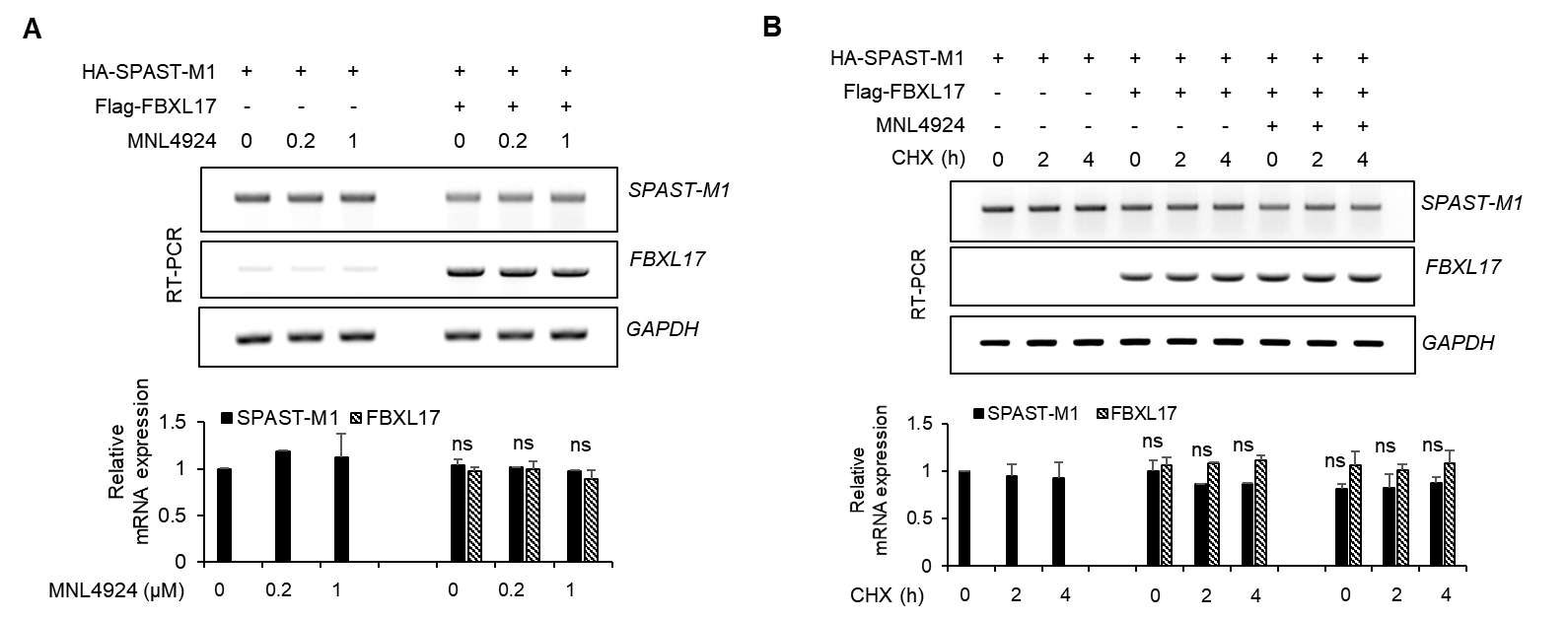


**Supplementary Figure 8.** (A) mRNA expression was analyzed and quantified using RT-PCR under the conditions shown in Figure 5B. (B) mRNA expression was analyzed using RT-PCR under the conditions shown in Figure 5C and quantified. Data are mean ± standard deviation. (*p <0.05; **p < 0.01; ns, not significant)


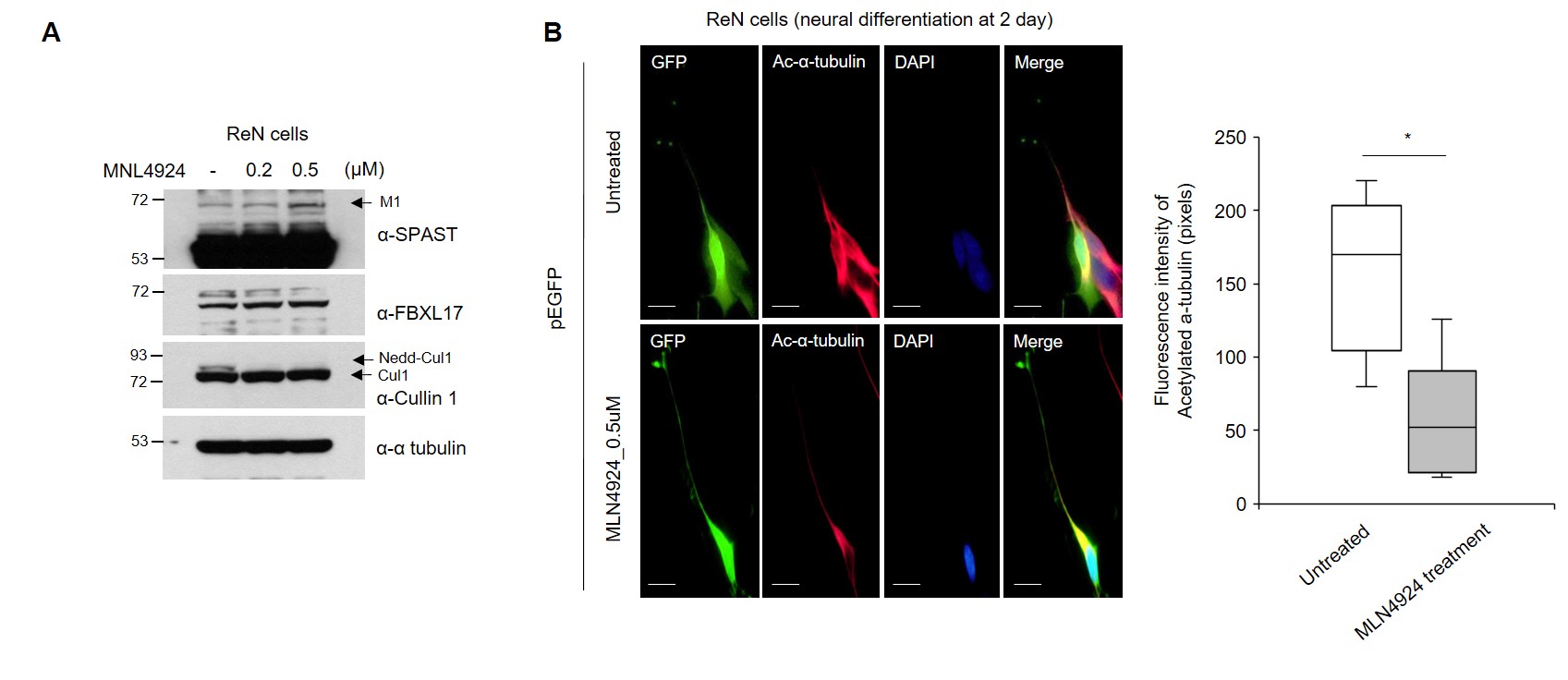


**Supplementary Figure 9.** (A) Rencell CX cells were treated with 0.2, 0.5 μM MLN4924 for 2 d, and analyzed using western blotting with the indicated antibodies. (B) After transfection with pEGFP plasmid, the cells were differentiated for 2 d in the presence or absence of 0.5 μM MLN4924 and stained with GFP and acetylated α-tubulin antibodies. Fluorescence intensities of acetylated α-tubulin quantified with ImageJ software. Data are mean ± standard deviation calculated from three replicates. Scale bar: 20 μm (*p < 0.05)


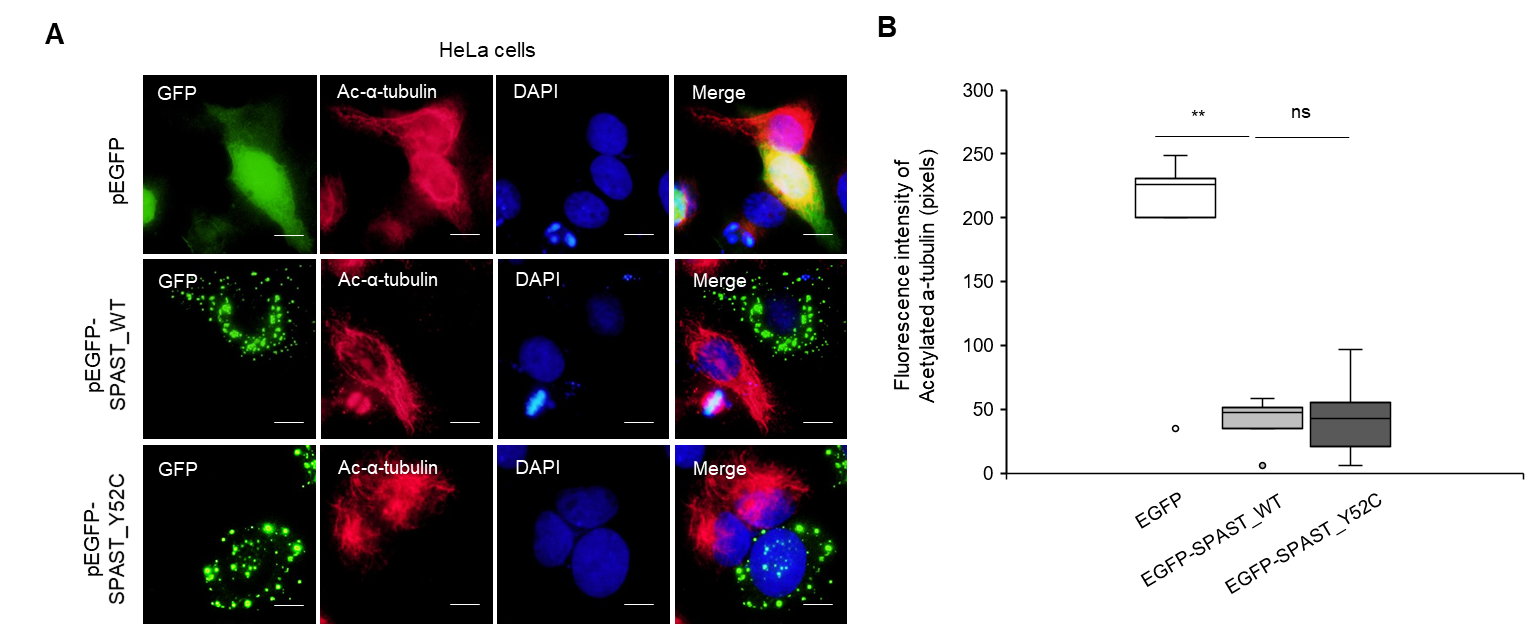


**Supplementary Figure 10.** (A) Immunostaining of GFP and Acetylated α-tubulin in HeLa cells transfected with GFP empty or GFP-SPAST-M1 plasmids, and (B) fluorescence intensities of acetylated α-tubulin quantified using ImageJ software. Data are mean ± standard deviation calculated from two replicates. Scale bar: 50 μm (**p < 0.01; ns, not significant)
