# Supplementary material for: FBXL17/spastin axis as a novel therapeutic target of hereditary spastic paraplegia
Source: Cell Biosci. 2022 Jul 22;12:110. doi: 10.1186/s13578-022-00851-1 (PMC9308218; doi:10.1186/s13578-022-00851-1)
Supplement: Supplementary file 2 — Additional file 2: Table S1. Antibody list used in this study. Table S2. Reagents list used in this study. Table S3. Plasmid constructs used in the study. Table S4. The primer list for conventional RT-PCR. Table S5. The primer list for RT-qPCR. Table S6. The predicted ubiquitination sites of SPAST. Table S7. The predicted CK2 phosphorylation sites of SPAST. Table S8. SPAST exon 1 mutation and clinical summary found in HSP patients. [file 13578_2022_851_MOESM2_ESM.docx]

**Supplementary tables**

**Table lists**

**Supplementary Table 1.** Antibody list used in this study

**Supplementary Table 2.** Reagents list used in this study

**Supplementary Table 3.** Plasmid constructs used in the study

**Supplementary Table 4.** The primer list for conventional RT-PCR

**Supplementary Table 5.** The primer list for RT-qPCR

**Supplementary Table 6.** The predicted ubiquitination sites of SPAST

**Supplementary Table 7.** The predicted CK2 phosphorylation sites of SPAST

**Supplementary Table 8.** SPAST exon 1 mutation and clinical summary found in HSP patients.

Supplementary Table 1. Antibody list used in this study (continued)

| **Antibodies** | **Company** | **Catalogue No.** | **Clone No.** | **Dilution for western blot** | **Dilution for IF** |
| --- | --- | --- | --- | --- | --- |
| **Primary Antibodies** | | | | | |
| β actin | Sigma-Aldrich | A1978 | AC-15 | 1:5000 | NA |
| Flag | Sigma-Aldrich | F3165 | M2 | 1:5000 | NA |
| Skp 1 | Santa-Cruz | sc5281 | H-6 | 1:2000 | NA |
| Cullin 1 | Santa-Cruz | sc17775 | D-5 | 1:2000 | NA |
| Rbx 1 | Santa-Cruz | sc393640 | E-11 | 1:2000 | NA |
| GST | Santa-Cruz | sc-138 | B-14 | 1:2000 | NA |
| HA probe | Santa-Cruz | sc7392 | F-7 | 1:2000 | NA |
| SPAST | RevMAb Biosciences | 31-1232-00 | RM346 | 1:2000 | 1:200 |
| Histone H3 | Santa-Cruz | sc517576 | 1G1 | 1:5000 | NA |
| DCAF8 | Santa-Cruz | sc100898 | QS-5 | 1:2000 | NA |
| p53 | Santa-Cruz | sc-126 | DO-1 | 1:3000 | NA |
| GADD153 (CHOP) | Santa-Cruz | sc-793 | R-20 | 1:3000 | NA |
| PUMA α/β | Santa-Cruz | sc-374223 | G-3 | 1:2000 | NA |
| His tag | Cell signaling | #2365 | polyclonal | 1:3000 | NA |
| α-tubulin | Cell signaling | #2125 | 11H10 | 1:3000 | NA |
| Acetyl α-tubulin (Lys40) | Cell signaling | #5335 | D20G3 | NA | 1:500 |
| Phospho CK2-substrate | Cell signaling | #8738 | mAb mix | 1:2000 | 1:300 |
| CK2α | Cell signaling | #2656 | polyclonal | 1:2000 | NA |
| MAP2 | Cell signaling | #4542 | polyclonal | 1:2000 | 1:200 |
| ATF4 | Cell signaling | #11815 | D4B8 | 1:2000 | NA |
| GFAP | Cell signaling | #3670 | GA5 | 1:2000 | 1:200 |
| GFP | Roche | 1181446001 | Clone 7.1,13.1 | NA | 1:500 |
| Phospho CK2α | Abcam | ab119410 | polyclonal | 1:2000 | NA |
| Tau | Abcam | ab64193 | polyclonal | 1:2000 | 1:300 |
| Phospho Threonine | Abcam | ab218195 | EPR22006-23 | 1:2000 | NA |
| SPAST | Abcam | ab77144 | Sp 6C6 | 1:3000 | 1:100 |
| FBXL17 | Abcam | ab111683. | polyclonal | 1:3000 | 1:100 |

*NA; not applicable

Supplementary Table 1. Antibody list used in this study

| **Antibodies** | **Company** | **Catalogue No.** | **Dilution for western blot** | **Dilution for IF** |
| --- | --- | --- | --- | --- |
| **Secondary Antibodies** | | | | |
| m-IgGκ BP-HRP | Santa-Cruz | sc516102 | 1:10000 | NA |
| Anti-rabbit IgG-HRP | Cell signaling | #7074 | 1:10000 | NA |
| Donkey anti-Mouse IgG, Alexa Fluor 488 | Invitrogen | A21202 | NA | 1:1000 |
| Goat anti-Rabbit IgG, Alexa Fluor 546 | Invitrogen | A11010 | NA | 1:1000 |

*NA; not applicable

Supplementary Table 2. Reagents list used in this study

| **Reagents** | **Company** | **Catalogue No.** | **Working conc.** |
| --- | --- | --- | --- |
| cOmplete™, Mini Protease Inhibitor Cocktail | Roche | 11836153001 | 1 tablet in 10 ml |
| isopropyl-b-D-thiogalactoside  (IPTG) | Sigma-Aldrich | I6758 | 0.5~1 mM |
| Phenylmethanesulfonyl fluoride  (PMSF) | Roche | 10837091001 | 1 mM |
| MG132 | Sigma-Aldrich | C2211 | 10 μM |
| Cycloheximide | Sigma-Aldrich | C0934 | 50 μg/ml |
| MLN4924  (Pevonedistat) | Med Chem Express | HY-70062 | 0.2~5 μM |
| CX-4945  (Silmitasertib) | Selleckchem | S2248 | 5 μM |
| Calyculin A | Cell signaling | #9902 | 10 nM |
| Paclitaxel | Sigma-Aldrich | T7191 | 200 nM |
| Anti-FLAG M2 Magnetic Beads | Sigma-Aldrich | M8823 | 5~10 μl/reaction |
| Anti-HA-magnetic beads | Thermo Fisher Scientific | 13464229 | 5~10 μl/reaction |
| Pierce™ Protein A Magnetic Beads | Thermo Fisher Scientific | 88845 | 15 μl/reaction |
| Ni-NTA agarose | QIAGEN | Ni-NTA resin | - |
| Glutathione Sepharose® 4B | Sigma-Aldrich | GE17-0756-01 | - |
| DAPI solution | BD Biosciences | #564907 | 0.1 μg/ml |
| [γ32P]ATP | perkinelmer | NEG002A250UC | 10 μCi/reaction |
| epidermal growth factor  (EGF) | Peprotech | AF-100-15 | 20 ng/ml |
| basic fibroblast growth factor  (bFGF) | Peprotech | 100-18B | 20 ng/ml |
| Forskolin | Sigma-Aldrich | F3917 | 10 μM |
| brain-derived neurotrophic factor  (BDNF) | Peprotech | 450-02 | 20 ng/ml |
| Retinoic acid | Sigma-Aldrich | R2625 | 5 μM |
| ER tracker dye | Thermo Fisher Scientific | E34250 | 1 μM |

Supplementary Table 3. Plasmid constructs used in the study (continued)

| **Plasmid** | **Construction** | **Source or Reference** |
| --- | --- | --- |
| pFlag-SPAST-M1 | SPAST fragment (1-616a.a) was amplified by RT-PCR using cDNA from HEK293T cells and subcloned into the BamHI/ HindIII sites of pCMV-tag2B | Lim, et al, 2018^1)^ |
| pFlag-SPAST-M87 | SPAST fragment (87-616a.a) was amplified by RT-PCR using cDNA from HEK293T cells subcloned into the BamHI/ HindIII sites of pCMV-tag2B | Lim, et al, 2018^1)^ |
| pFlag-SPAST-ΔNT2 | SPAST fragment (151-616a.a) was amplified and subcloned into the BamHI/ HindIII sites of pCMV-tag2B | This study |
| pFlag-SPAST-ΔNT3 | SPAST fragment (301-616a.a) was amplified and subcloned into the BamHI/ HindIII sites of pCMV-tag2B | This study |
| pFlag-SPAST- NT | SPAST fragment (1-250a.a) was amplified and subcloned into the BamHI/ HindIII sites of pCMV-tag2B | This study |
| pGEX4T1-SPAST-M1 | amplified from pFlag-SPAST-M1 and subcloned into the BamHI/ SalI sites of pGEX4T1 | This study |
| pHA-SPAST-M1 | SPAST-M1 containing Ha-tag was amplified from pFlag-SPAST-M1 and subcloned into BamHI/HindIII sites of pShuttle-CMV | Lim, et al, 2018^1)^ |
| pFlag-SPAST-K206R | The lysine to arginine mutation at position in the p206 of SPAST-M1 was amplified from pFlag-SPAST-M1 using primers with the desired mutation, and subcloned into the BamHI/ HindIII sites of pCMV-tag2B | This study |
| pFlag-SPAST-K234R | The lysine to arginine mutation at position in the p234 of SPAST-M1 was amplified from pFlag-SPAST-M1 using primers with the desired mutation, and subcloned into the BamHI/ HindIII sites of pCMV-tag2B | This study |
| pFlag-SPAST-K236R | The lysine to arginine mutation at position in the p236 of SPAST-M1 was amplified from pFlag-SPAST-M1 using primers with the desired mutation, and subcloned into the BamHI/ HindIII sites of pCMV-tag2B | This study |
| pFlag-SPAST-K254R | The lysine to arginine mutation at position in the p254 of SPAST-M1 was amplified from pFlag-SPAST-M1 using primers with the desired mutation, and subcloned into the BamHI/ HindIII sites of pCMV-tag2B | This study |
| pFlag-SPAST-K279R | The lysine to arginine mutation at position in the p279 of SPAST-M1 was amplified from pFlag-SPAST-M1 using primers with the desired mutation, and subcloned into the BamHI/ HindIII sites of pCMV-tag2B | This study |
| pFlag-SPAST-K421R | The lysine to arginine mutation at position in the p421 of SPAST-M1 was amplified from pFlag-SPAST-M1 using primers with the desired mutation, and subcloned into the BamHI/ HindIII sites of pCMV-tag2B | This study |
| pFlag-SPAST-K519R | The lysine to arginine mutation at position in the p519 of SPAST-M1 was amplified from pFlag-SPAST-M1 using primers with the desired mutation, and subcloned into the BamHI/ HindIII sites of pCMV-tag2B | This study |
| pFlag-SPAST-K532R | The lysine to arginine mutation at position in the p532 of SPAST-M1 was amplified from pFlag-SPAST-M1 using primers with the desired mutation, and subcloned into the BamHI/ HindIII sites of pCMV-tag2B | This study |
| pFlag-SPAST-K554R | The lysine to arginine mutation at position in the p554 of SPAST-M1 was amplified from pFlag-SPAST-M1 using primers with the desired mutation, and subcloned into the BamHI/ HindIII sites of pCMV-tag2B | This study |

Supplementary Table 3. Plasmid constructs used in the study (continued)

| **Plasmid** | **Construction** | **Source or Reference** |
| --- | --- | --- |
| pFlag-SPAST-T530A | The threonine to alanine mutation at position in the p530 of SPAST-M1 was amplified from pFlag-SPAST-M1 using primers with the desired mutation, and subcloned into the BamHI/ HindIII sites of pCMV-tag2B | This study |
| pFlag-SPAST-S545A | The serine to alanine mutation at position in the p545 of SPAST-M1 was amplified from pFlag-SPAST-M1 using primers with the desired mutation, and subcloned into the BamHI/ HindIII sites of pCMV-tag2B | This study |
| pFlag-SPAST-S547A | The serine to alanine mutation at position in the p547 of SPAST-M1 was amplified from pFlag-SPAST-M1 using primers with the desired mutation, and subcloned into the BamHI/ HindIII sites of pCMV-tag2B | This study |
| pFlag-SPAST-S573A | The serine to alanine mutation at position in the p573 of SPAST-M1 was amplified from pFlag-SPAST-M1, and subcloned into the BamHI/ HindIII sites of pCMV-tag2B | This study |
| pFlag-SPAST-S583A | The serine to alanine mutation at position in the p583 of SPAST-M1 was amplified from pFlag-SPAST-M1 using primers with the desired mutation, and subcloned into the BamHI/ HindIII sites of pCMV-tag2B | This study |
| pFlag-SPAST-Y52C | The tyrosine to cysteine mutation at position in the p52 of SPAST-M1 was amplified from pFlag-SPAST-M1 using primers with the desired mutation, and subcloned into the BamHI/ HindIII sites of pCMV-tag2B | Lim, et al, 2018^1)^ |
| pEGFP-SPAST-M1 | amplified from pFlag-SPAST-M1 and subcloned into the HindIII / BamHI sites of pEGFP-C2 | Lim, et al, 2018^1)^ |
| pEGFP-SPAST-M87 | amplified from pFlag-SPAST-M87 and subcloned into the HindIII / BamHI sites of pEGFP-C2 | Lim, et al, 2018^1)^ |
| pEGFP-SPAST-Y52C | amplified from pFlag-SPAST-Y52C and subcloned into the HindIII / BamHI sites of pEGFP-C2 | This study |
| pFlag-FBXL17 | FBXL17 was amplified by RT-PCR using cDNA from MCF7 cells and subcloned into the BamHI/ HindIII sites of pCMV-tag2B | This study |
| pEBG-FBXL17-ΔNT1 | FBXL17 fragment (318-701a.a) was amplified from pFlag-FBXL17 and subcloned into the BamHI/ NotI sites of pEBG | This study |
| pEBG-FBXL17-ΔNT2 | FBXL17 fragment (371-701a.a) was amplified from pFlag-FBXL17 and subcloned into the BamHI/ NotI sites of pEBG | This study |
| pEBG-FBXL17-ΔNT3 | FBXL17 fragment (471-701a.a) was amplified from pFlag-FBXL17 and subcloned into the BamHI/ NotI sites of pEBG | This study |
| pEBG-FBXL17-ΔNT4 | FBXL17 fragment (571-701a.a) was amplified from pFlag-FBXL17 and subcloned into the BamHI/ NotI sites of pEBG | This study |
| pEBG-FBXL17-F box | FBXL17 fragment (318-370a.a) was amplified from pFlag-FBXL17 and subcloned into the BamHI/ NotI sites of pEBG | This study |
| pET28a-FBXL17-ΔNT1 | BamHI/ NotI fragment (FBXL17_318-701a.a) of pEBG-FBXL17-ΔNT1 replaced into the BamHI/ NotI sites of pET28a | This study |

Supplementary Table 3. Plasmid constructs used in the study

| **Plasmid** | **Construction** | **Source or Reference** |
| --- | --- | --- |
| pFlag-FBXL2 | FBXL2 was amplified by RT-PCR using cDNA from HEK293 cells and subcloned into the EcoRI/ApaI sites of pCMV-tag2B | This study |
| pFlag-FBXL3 | FBXL3 was amplified by RT-PCR using cDNA from HEK293 cells and subcloned into the BamHI/ XhoI sites of pCMV-tag2B | This study |
| pFlag-FBXL5 | FBXL5 was amplified by RT-PCR using cDNA from HEK293 cells and subcloned into the BamHI/ XhoI sites of pCMV-tag2B | This study |
| pFlag-FBXL12 | FBXL12 was amplified by RT-PCR using cDNA from HEK293 cells and subcloned into the EcoRV/ApaI sites of pCMV-tag2B | This study |
| pFlag-FBXL15 | FBXL15 was amplified by RT-PCR using cDNA from HEK293 cells and subcloned into the BamHI/ HindIII sites of pCMV-tag2B | This study |
| pFlag-CK2α | CK2α was amplified by RT-PCR using cDNA from HeLa cells and subcloned into the HindIII /BamHI sites of pFlag-CMV2 | This study |
| pFlag-CK2β | CK2β was amplified by RT-PCR using cDNA from HeLa cells and subcloned into the HindIII /BamHI sites of pFlag-CMV2 | This study |
| pGEX4T1-KLHL12 | KLHL12 was amplified by RT-PCR using cDNA from HEK293 cells and subcloned into the BamHI/ SalI sites of pGEX4T1 | This study |
| pGEX4T1-SPAST-K554R | amplified from pFlag-SPAST-K554R and subcloned into the BamHI/ SalI sites of pGEX4T1 | This study |
| pFlag-SPAST-T530D, S545D,S547D | The threonine and serine to aspartic acid mutation at position in the p530, 545, and 547 of SPAST-M1 was amplified from pFlag-SPAST-M1 using primers with the desired mutation through PCR-based site directed mutagenesis | This study |
| Lentivirus-SPAST-M1_WT | amplified from pFlag-SPAST and subcloned into the NotI/BamHI sites of pLVX-EF1a-IRES-ZsGreen1 | This study |
| Lentivirus-SPAST-M1_Y52C | amplified from pFlag-SPAST-Y52C and subcloned into the NotI/BamHI sites of pLVX-EF1a-IRES-ZsGreen1 | This study |
| CK2β CRISPR/Cas9 | The CK2β CRISPR/Cas9 Plasmids construct was purchased from Santa-cruz.(Plasmid sc-401684) | This study |
| CK2β HDR | The CK2β HDR Plasmids construct was purchased from Santa-cruz.  (Plasmid sc-401684-HDR) | This study |

1) Lim JH, Kang HM, Jung H-R, Kim D-S, Noh KH, Chang TK, Kim BJ, Sung DH, Cho H-S, and Chung K-S. Missense mutation of SPAST protein (I344K) results in loss of ATPase activity and prolonged the half-life, implicated in autosomal dominant hereditary spastic paraplegia. *Biochimica et Biophysica Acta (BBA)-Molecular Basis of Disease.* 2018;1864(10):3221-33.

Supplementary Table 4. The primer list for conventional RT-PCR

| **Gene name** | **Species** | **Forward primer**  **(5'-3')** | **Reverse primer**  **(5'-3')** | **AT (℃)** |
| --- | --- | --- | --- | --- |
| *Nestin* | Mus musculus | CTC GAG CAG GAA GTG GTA GG | TTG GGA CCA GGG ACT GTT AG | 57 |
| *Emx2* | Mus musculus | ACC TTC TAC CCC TGG CTC AT | TCT CCA CCG GTT AAT GTG GT | 57 |
| *Dcx* | Mus musculus | TTG GAC ATT TTG ACG AAC GA | CCC TTC TTC CAG TTC ATC CA | 55 |
| *Tbr1* | Mus musculus | ACA GCC TGC TGT CCA ACT CT | TGC ATA TAG ACC CGG TTT CC | 55 |
| *NeuN* | Mus musculus | GCA CAG ACT CAT CCT GAG CA | GGC TGT GGC ATT ATT GAC CT | 55 |
| *Fbxl17* | Mus musculus | GGG CTC AAG ATG CAG AGA AC | CAA TGA CCT CCA CAC ACC TG | 55 |
| *Spast-M1* | Mus musculus | GGA CGA CGG AAG AAG AAA GG | GAA GAG GAG CCC CAG GTG | 57 |
| *Gapdh* | Mus musculus | AAC TTT GGC ATT GTG GAA GG | ACA CAT TGG GGG TAG GAA CA | 55 |
| *FBXL17* | Homo sapiens | GGC TTC ATG GGT TGT TCA GT | CAA TGA CCT CCA CAC ACC TG | 55 |
| *SPAST-M1* | Homo sapiens | GAC GAG GGA AGA AGA AAG G | AAC AGC GGG TAG GAG AAA TA | 56 |
| *MAP2* | Homo sapiens | GAACCAGCAGAATTCAGAG | GCTGCCTCTTCTACTTCAAA | 55 |
| *GAPDH* | Homo sapiens | GAG TCA ACG GAT TTG GTC GT | TTG ATT TTG GAG GGA TCT CG | 55 |

Supplementary Table 5. The primer list for RT-qPCR

| **Gene name** | **Species** | **Forward primer**  **(5'-3')** | **Reverse primer**  **(5'-3')** |
| --- | --- | --- | --- |
| *SPAST* | Homo sapiens | TGG ACG AGG GAA GAA GAA AG | ACA GCG GGT AGG AGA AAT AG |
| *CK2α* | Homo sapiens | GGC TTA CTG CAA GAG AGG CA | ATG AAC CCA TTC GAG CCT GG |
| *CK2β* | Homo sapiens | AAG ACA ACC CCA ACC AGA G | CAC GAG GAC AGT AAC CAA AG |
| *GAPDH* | Homo sapiens | GAGTCAACGGATTTGGTCGT | TTGATTTTGGAGGGATCTCG |

Supplementary Table 6. The predicted ubiquitination sites of SPAST

| **Residue** | **sequences** | **Score** | **ubiquitinated** |
| --- | --- | --- | --- |
| K206 | QPVLPFS**K**SQTDVYN | 1.20 | Medium confidence |
| K234 | SESGAVP**K**RKDPLTH | 2.79 | High confidence |
| K236 | SGAVPKR**K**DPLTHTS | 1.48 | Medium confidence |
| K254 | PRSKTVM**K**TGSAGLS | 1.58 | Medium confidence |
| K279 | LSMVSGV**K**QGSGPAP | 0.85 | Medium confidence |
| K421 | KYVGEGE**K**LVRALFA | 1.63 | Medium confidence |
| K519 | ETRLLLL**K**NLLCKQG | 0.89 | Medium confidence |
| K532 | QGSPLTQ**K**ELAQLAR | 2.35 | High confidence |
| K554 | SDLTALA**K**DAALGPI | 1.55 | Medium confidence |

*BDM-PUB server: http://bdmpub.biocuckoo.org/

Supplementary Table 7. The predicted CK2 phosphorylation sites of SPAST

| **Position** | **Code** | **Kinase** | **Peptide** | **Score** | **Cutoff** |
| --- | --- | --- | --- | --- | --- |
| 530 | T | CMGC/CK2 | CKQGSPL**T**QKELAQL | 1.955 | 1.074 |
| 545 | S | CMGC/CK2/CK2a1 | ARMTDGY**S**GSDLTAL | 4.754 | 3.086 |
| 547 | S | CMGC/CK2/CK2a1 | MTDGYSG**S**DLTALAK | 3.369 | 3.086 |
| 573 | S | CMGC/CK2/CK2a1 | PEQVKNM**S**ASEMRNI | 3.244 | 3.086 |
| 583 | S | CMGC/CK2/CK2a1 | EMRNIRL**S**DFTESLK | 3.879 | 3.086 |

*GPS (Group-based prediction system) 5.0 software: <http://gps.biocuckoo.cn/>

Supplementary Table 8. SPAST exon 1 mutation and clinical summary found in HSP patients.

| **location** | **Mutation** | **Amino-acid change** | **Family history** | **Age at onset if index patient** | **Clinical phenotype** | **reference** | **Ethnicity** |
| --- | --- | --- | --- | --- | --- | --- | --- |
| Exon1 | c.139 A>T | p.K47X,Term47 | AD | 40 | pure | Ishiura, et al, 2014^1)^ | Japanese |
| Exon1 | c.155 A>G | p.Y52C | Sporadic with consanguineous parents | 49 | pure | Ishiura, et al, 2014^1)^ | Japanese |
| Exon1 | c.283_323 del | p.A95fs | AD | 40 | pure | Ishiura, et al, 2014^1)^ | Japanese |
| Exon1 | c.343_352 dup | p.V118fs | AD | 35 | pure | Ishiura, et al, 2014^1)^ | Japanese |
| Exon1 | c.284delC | p.Ala95Argfs,Term65 | AD | - | - | Kadnikova, et al, 2019^2)^ | Russian |
| Exon1 | c.256 C>T | p.S44L | AD | 60 | pure | Lindsey, et al, 2000^3)^ | British |
| Exon1 | c.411delG | p.96-159fs,Term160 | AD | 2-33  (for seven affected members from two generation of this family) | pure | Lindsey, et al, 2000^3)^ | British |
| Exon1 | c.465G>T | p.E114X,Term114 | AD | 39-62  (for four of affected individuals) | pure | Svenson, et al, 2001^4)^ | British |

Abbreviations: HSP; Hereditary spastic paraplegia, del; deletion, fs; frameshift, dup; duplication, Term; Termination, AD; Autosomal dominant

1) Ishiura, Hiroyuki, et al. "Molecular epidemiology and clinical spectrum of hereditary spastic paraplegia in the Japanese population based on comprehensive mutational analyses." Journal of human genetics 59.3 (2014): 163-172.

2) Kdnikova, V. A., et al. "Mutational spectrum of Spast (Spg4) and Atl1 (Spg3a) genes in Russian patients with hereditary spastic paraplegia." Scientific reports 9.1 (2019): 1-8.

3) Lindsey, J. C. et al. Mutation analysis of the spastin gene (SPG4) in patients with hereditary spastic paraparesis. J. Med. Genet. 37, 759–765 (2000).

4) Svenson, Ingrid K., et al. "Identification and expression analysis of spastin gene mutations in hereditary spastic paraplegia." *The American Journal f Human Genetics* 68.5 (2001): 1077-1085.
